# Propofol emulsification in Intralipid and SMOFlipid: A promising alternative in response to future shortages

**DOI:** 10.1371/journal.pone.0331651

**Published:** 2025-09-08

**Authors:** Maxime Murphy, Mihaela Friciu, Valérie Gaëlle Roullin, Grégoire Leclair

**Affiliations:** Plateforme de Biopharmacie, Université de Montréal, Montréal, Quebec, Canada; Sapienza University of Rome: Universita degli Studi di Roma La Sapienza, ITALY

## Abstract

**Introduction:**

The Covid-19 pandemic has intensified shortages in various pharmaceutical products, notably injectable propofol in lipid emulsion form. Its demand surged sharply due to its critical role in intubating patients with respiratory distress during the pandemic, exposing vulnerabilities in the supply chain for this essential product.

**Objectives:**

This project aims to develop an alternative formulation to commercially available propofol products and to evaluate its stability through a detailed study.

**Methods:**

Two lipid emulsions commonly used for intravenous nutrition, Intralipid 20% and SMOFlipid 20%, were selected as diluents for pure propofol due to their composition’s similarity to DIPRIVAN, the standard propofol product. We developed and validated an HPLC method for quantifying propofol and employed an optimized laser diffraction technique to measure particle size. Additionally, we assessed the pH of the formulations.

**Results:**

The preparation method demonstrated repeatability and homogeneity. Stability studies revealed that the propofol concentrations remained close to the target of 10 mg/mL (1%). Although particle sizes were larger compared to DIPRIVAN, they were consistent with those of the lipid emulsions before propofol addition. The pH of the formulations remained stable throughout the study period.

**Conclusions:**

The developed propofol emulsion formulations met USP standards for all tested parameters over a period of at least 7 days, indicating that these alternatives are a viable and stable substitute for commercial propofol products.

## Introduction

**Propofol** is a small hydrophobic molecule widely used as an anesthetic to induce general anesthesia for prolonged and invasive surgeries. Additionally, it serves as a rapid-onset injectable sedative in hospital settings. Due to its rapid metabolism in vivo, propofol is often administered via continuous infusion during procedures [1,2]. The COVID-19 pandemic has exposed significant weaknesses in the supply chains of various pharmaceutical products, leading to shortages driven by increased demand within healthcare systems. Propofol emulsion was particularly affected by these shortages, largely due to its critical role in facilitating invasive mechanical ventilation for patients with acute respiratory distress syndrome [3,4]. Therefore, it is crucial to develop alternative production methods for injectable propofol to mitigate the risk of future shortages.

In response to these shortages, Médicament Québec, a government agency in Quebec, Canada, is working to strengthen the supply chains for certain pharmaceuticals by funding innovative projects aimed at addressing the scarcity of critical drugs. The first drug targeted by this agency was injectable propofol emulsion.

Some products used for intravenous nutrition in hospitals have compositions similar to the original propofol formulation, DIPRIVAN. For example, Intralipid 20% and SMOFlipid 20%, both oil-in-water lipid emulsions originally marketed for intravenous nutrition, could potentially serve as diluents for pure propofol [1,5,6]. It is worth investigating whether these products can be used to formulate injectable propofol. Intralipid 20% has previously been used as a vehicle for various active substances, including paclitaxel, ciprofloxacin, amphotericin B, and nystatin [7–10]. Although this method was successful for these substances, challenges were noted, particularly with amphotericin B and nystatin due to their large molecular size and low lipophilicity, which required prolonged mixing to achieve effective encapsulation [9,10].

In contrast, propofol is a small, highly lipophilic molecule, which suggests it may be well-suited for this formulation technique [1]. Additionally, it would be beneficial to explore whether SMOFlipid 20% could serve as an alternative to Intralipid in case of shortages.

Recent studies have explored the dilution of propofol in intravenous lipid formulations, with varying results. Cèbe et al. attempted to formulate propofol at a concentration of 2% in Intralipid 20% through simple dilution but found the product to be unstable, with phase separation between the oil (propofol) and lipid (Intralipid) phases, which could be hazardous for human administration [11]. Conversely, Rooimans et al. used SMOFlipid 20% with 0% to 10% of propofol content and found their formulation to be stable without phase separation when using a propofol concentration lower than 2%, suggesting it could be a viable alternative to the commercial products during shortages [12]. We suggest multiple improvements compared to those two articles by optimizing preparation so that it can be used daily in hospitals, but also by testing the method on two different lipid emulsions in case of a shortage of such products.

This article aims to evaluate whether a simple dilution of pure propofol in Intralipid 20% and SMOFlipid 20% at a low concentration (1%) can produce an injectable propofol emulsion that meets USP standards for emergency use. The usage of a gentle manual mixing technique and a different sterilization process should resolve the problems observed in Cèbe et al. article. It would also prove that complicated mixing techniques used in Rooimans et al. that are not suitable for hospital usage are not necessary to produce a norm complying preparation. A stability study will also be conducted to assess the viability of these formulations.

## Materials and methods

An innovative method for synthesizing pure propofol was developed by the Department of Continuous Flow Synthesis at the Faculty of Chemistry, University of Montreal, during the Covid-19 pandemic. This synthesis method is similar to the one described by Mougeot et al. and provided us with a propofol of 99.1% purity for our project [13]. Additionally, the purity of the synthetized propofol was confirmed by using a propofol standard sourced from *Toronto Research Chemical*.

### Propofol compounded formulation preparation

The formulations tested in this article involve a straightforward dilution of propofol in two different lipid vehicles: Intralipid 20% (IL) and SMOFlipid 20% (SMOF). The target concentration of propofol is 10 mg/mL, which matches the concentration of the commercially available ready-to-use formulation of this molecule in Canada [1].

A volume of 530 µL of 99.1% pure propofol previously sterilized with a 0.22 µm PVDF filter, is measured with a 1 mL graduated plastic syringe and added to a sterile amber 60 mL glass injection vial. This volume (530 µL) corresponds to an approximate mass of 505 mg of pure propofol (≈509.5 mg of 99.1% pure propofol). This volume was determined during method development by measuring the mass of a 1 mL syringe before and after the addition of different volume of propofol (between 500–550 µL). 530 µL was the volume that was producing the closest mass to 509.5 mg. To ensure accuracy, the exact mass of propofol added in vials is determined by weighing the vial before and after the addition using an analytical balance. This procedure ensures that the mass of propofol in each vial is consistent and reproducible.

Thereafter, 50 mL of the selected lipid emulsion is measured using a 60 mL plastic syringe and added to the propofol-containing vial, resulting in a final emulsion volume of approximately 50.5 mL with a propofol concentration of 10 mg/mL (i.e., 505 mg of pure propofol). This step is performed under a sterile hood using aseptic technique to ensure the sterility of the final product is maintained.

The final step involves gentle manual mixing of the two components directly in the glass vial. This mixing consists of repeated manual inversions and low-intensity shaking by hand for approximately 1 minute. The mixing can be visually monitored by checking for any remaining yellowish oil droplets (unemulsified propofol) to confirm that the propofol has been fully emulsified into the lipid medium.

### Content uniformity

Uniformity of content is assessed by taking 10 samples of 1 mL from various positions within a vial containing the propofol-lipid mixture, prepared according to the method described above. The samples are collected after a few seconds of manual mixing. This procedure is performed twice: once with Intralipid and once with SMOFlipid. Ratios are then calculated in accordance with the method outlined in USP Chapter <905> [14], using propofol quantification results obtained via the High-Performance Liquid Chromatography (HPLC) method developed in this study. The predefined limits for these ratios are based on the average percentage of nominal content and the standard deviation of these values.

### Preparation method validation

Evaluating the repeatability and accuracy of the preparation method is crucial to ensure that the finished products consistently have exact and reproducible content. Therefore, the content results from three different preparations (triplicates) in each lipid emulsion were analyzed. The percentage of the mean nominal content, along with the standard deviation of this mean, is determined to verify that the preparations contain the desired amount of propofol.

### Stability study

To conduct the stability study, a total of four preparations were made using the aforementioned method: two with Intralipid 20% and two with SMOFlipid 20%. Each 50.5 mL formulation was then divided equally into three 30 mL amber glass injection vials, resulting in a total of 6 vials per type of diluent. Half of the vials were stored in a refrigerator at 4°C, while the other half were kept at ambient temperature (25°C). The entire batch was stored upside down to simulate a worst-case degradation scenario, ensuring that the formulations were in contact with all components of the vials, including the rubber stopper.

At each sampling time, a 1 mL sample of the formulated propofol was taken under a sterile hood using a 1 mL plastic syringe for particle size, content, and pH measurements. A positive aspect of the formulation strategy tested in this study is that the finished product will be a ready to use vial containing pure propofol. The stability of the preparations does not need to be established over a long period, as clinical practice requires injection within a few hours of reconstitution. Nonetheless, to ensure thorough evaluation, the stability study was conducted over 7 days, which is a longer period than typically required in hospitals if this technique proves effective.

#### Particle size assessment.

An important aspect of quality control for the resulting formulations is measuring lipid particle size. For this purpose, two techniques were used. The first technique is laser diffraction by using the Beckman Coulter LS 13 320 with the Universal Liquid Module. To prepare the analysis samples, 100 µL of each of the 12 propofol-lipid formulations were diluted in a 15-mL plastic tube containing 10 mL of water. After manual mixing, the diluted product was added drop by drop until a PIDS (Polarization Intensity Differential Scattering) obscuration of 40% was achieved on the LS Coulter. Obscuration represents the extent to which the light beam (laser) is blocked by the suspended particles in the dilution. Once the target obscuration was reached, a measurement sequence consisting of three analyses, each lasting a total of 270 seconds, was initiated. This sequence was repeated for each of the twelve samples. Afterwards, to ensure no large globule were created, dynamic light scattering (DLS) was the second technique used. This technique is sensitive to bigger particle sizes and supports the results obtained by laser diffraction. This test was done in a different stability study on n = 1 of each formulation. To prepare DLS analysis samples, 10 µL of each preparation [4] were diluted in 10 mL of water. They were then analyzed with a Brookhaven NanoBrook Omni particle analyzer by three consecutive measurements of 3 minutes each. These techniques address the criteria required to meet USP Chapter <729> on particle size assessment [15].

#### HPLC method development.

The propofol content of the samples was measured using a high-performance liquid chromatography (HPLC) method that has been developed, optimized, and validated to meet USP requirements. The HPLC system (Prominence UFLC, Shimadzu) was equipped with an LC-20AD binary pump operating at a flow rate of 0.75 mL/min, a DGU-20A5 solvent degasser, an SPD-M20A multiple-wavelength photodiode array detector set at 272 nm for propofol, an SIL-20 AC HT refrigerated autosampler at 5°C, and a CTO-20 AC column oven at 40°C. The final method was isocratic, with a mobile phase consisting of 60% 20 mM potassium phosphate buffer (KH₂PO₄) at pH 2.5 and 40% acetonitrile (ACN). The analysis was conducted using a Phenomenex Kinetex 5 µm XB-C18 100 x 3.0 mm column. The injection volume was optimized to 10 µL. Full details of the method are provided in Table 1.

**Table 1:** Optimized and validated Propofol HPLC method conditions.

HPLC	Shimadzu Prominence DGU-20A
**Product, **λ	Propofol, λ = 272nm
**Detector**	PDA
**Mobile phase**	Mobile phase A: ACN Mobile phase B: Phosphate Buffer (KH_2_PO_4_) 20mM at pH = 2.5
**Mode**	Isocratic (40% Phase A + 60% Phase B)
**Flow rate**	0.6 mL/min
**Temperature of the column**	40 °C
**Volume of injection**	10 µL
**Column**	Phenomenex Kinetex XB-C18 (3.0 × 100 mm, 5 µm, 100 Å) *P/N*: 00D-4605-Y0 *S/N*: H22-058645 *B/N*: 5705−0098
**Retention time**	Propofol – 12.5 min

#### HPLC method validation.

Validation of the HPLC method was carried out based on ICH Q2 (R1) criteria [16], as well as other predefined method validation criteria commonly used. The principal validation criteria are intra and inter-day variability/repeatability, linearity, precision/accuracy, and specificity. Intra and inter-day variability is tested by injecting in triplicate a calibration curve during the same day and during three different days. This test is also used to assess the linearity, accuracy and precision of the method. The specificity is tested by injecting a sample containing only the analyte, a sample containing the principal degradation compounds mentioned in propofol USP chapter [17] and a sample containing a mix of the analyte and the same degradation compounds to ensure that no degradation products create a peak at the same retention time as the analyte.

#### Propofol extraction.

Since the samples are lipid emulsions, it is necessary to extract propofol through lipid solubilization. The solvent used for this extraction was optimized. When solubilization is not optimal, lipid emulsions produce a highly opaque solution. Conversely, when lipid solubilization is optimal, the solution becomes immediately transparent, showing a clear contrast. Therefore, the opacity of 9.9 mL of different solvent mixtures was visually assessed after the addition of 100 µL of lipid formulations to evaluate the effectiveness of the solubilization. The solvents tested in different ratios were acetonitrile, methanol and water.

#### Propofol content measurement.

For content measurement during the stability study, twelve extractions (one for each vial) were performed using the optimal extraction technique described in the *Results* section. 1 mL of each extraction was then transferred to an HPLC vial, which was subsequently analyzed by HPLC. The series of injections was initiated using the validated HPLC method outlined in Table 1.

The calibration curve was generated by diluting pure propofol directly in the solvent mixture used for extraction. Calibration samples were prepared in HPLC vials from a stock solution of propofol at 1 mg/mL, which was then diluted to obtain concentrations ranging from 80% to 120% of the content measured from the extractions of 100 µL of our preparations in 10 mL of the extraction solvent mixture. Details of the composition for each calibration sample are provided in S2 Table (S1 File).

The calibration curve used throughout the stability study was the same over the entire 7-day period, as inter-day variability was found to be negligible during method validation. However, a quality control sample representing 100% of the target content was injected daily to ensure the reproducibility of the method across different days.

#### pH measurement of preparations.

The third factor in evaluating the conformity of our preparation was pH. According to USP standards, the pH of injectable lipid emulsions must fall within the range of 4.5 to 8.5 [18]. To measure pH, a HANNA pH meter was used. The pH of each sample taken at each time point during the stability study was measured directly in the 1.5-mL plastic tube containing the propofol-lipid mixture, immediately after taking the 100 µL samples for particle size and content measurements. The pH meter was calibrated daily using pH 4.01 and 7.00 standard solutions before any measurements are taken.

## Results

### Content uniformity

Content uniformity was demonstrated for both formulations. As shown in Table 2, the ratios calculated according to USP Chapter <905 > are well below 15.0, confirming the uniformity of propofol content. The detected content ranges from 98.5% to 101.5% of the nominal value, with the ratio defined as k × s, where k = 2.4 and s represents the standard deviation of the content across the 10 samples [14].

**Table 2:** Uniformity of content of 2 propofol preparations, either in Intralipid 20% (IL) or SMOFlipid 20% (SMOF).

Sample name	Number of samples	Mean of % of nominal content	USP Ratio	USP Acceptance criteria [14]
Propofol-IL	10	101.2% ± 0.9%	2.1	Ratio < 15.0
Propofol-SMOF	10	101.0% ± 1.0%	2.5	

### Repeatability and accuracy of preparation method

The preparation method was found to be both accurate and reproducible (n = 3). As detailed in Table 3, the average detected content is very close to the target content, with a standard deviation of less than 1%.

**Table 3:** Accuracy of content with the preparation method developed for propofol in Intralipid 20% (IL) and SMOFlipid 20% (SMOF) (n = 3).

Preparation	% of nominal content			Mean (%)	Standard deviation (%)	Acceptance criteria
	Sample 1	Sample 2	Sample 3			
Propofol-IL	99.9%	99.4%	101.2%	100.2%	0.9%	Mean = [97%–103%]
Propofol-SMOF	101.3%	100.5%	101.0%	100.9%	0.4%	

### Propofol extraction

The optimal solvent mixture for solubilizing the lipids in Intralipid and SMOFlipid was identified as a 1:1 (v/v) mixture of methanol (MeOH) and acetonitrile (ACN). A total volume of 10 mL of this solvent mixture was sufficient to fully solubilize up to 200 µL of lipid emulsion containing 20% lipids.

During the stability study, a single-step extraction was performed by adding 9.9 mL of this solvent mixture to 100 µL of each propofol preparation. The mixture was then vortexed vigorously, and each extract was analyzed individually.

The extraction efficiency was determined to be 100.0 ± 0.3% when this method was applied to three independent preparations of propofol in SMOFlipid 20%. Due to the large dilution factor achieved during extraction, centrifugation to remove residual lipids was deemed unnecessary.

The theoretical concentration of propofol after extraction was 100 µg/mL, corresponding to the 100% standard point of the calibration curve used for quantification.

### Stability study

#### Particle size assessment.

Table 4 presents the mean particle sizes of the marketed products used in this study, reported as mean ± standard deviation. These results will serve as a reference for comparing our propofol formulations and assessing the impact of propofol addition on the particle size distribution of the lipids used (Intralipid and SMOFlipid). Note that a sample of DIPRIVAN, the original commercial propofol product, was also analyzed for comparison purposes.

**Table 4:** Average particle size of marketed reference products in triplicate measurements (n = 1).

Sample	Mean size (nm)
Intralipid 20%	325 ± 1
SMOFlipid 20%	266 ± 1
DIPRIVAN	203 ± 0

The particle size results for our formulations during the stability study were unequivocal. As shown in Table 5, the mean particle sizes, which must be below 500 nm according to USP Chapter <729> [15], are all well below this limit. The sizes reported represent the average (n = 3) mean particle sizes in our propofol formulations.

**Table 5:** Mean particle size of 12 propofol formulations in Intralipid 20% (IL) or SMOFlipid 20% (SMOF) (n = 3) kept under different temperature conditions.

Temperature	Sample name	Mean (n = 3) particle size (nm)					USP Acceptance criteria [15]
		t = 0	24h	48h	72h	7 days	
25^o^C	Prop-IL-25^o^C	324 ± 1	325 ± 1	323 ± 1	323 ± 1	325 ± 1	< 500 nm
	Prop-SMOF-25^o^C	267 ± 3	269 ± 2	269 ± 1	269 ± 2	268 ± 2	
4^o^C	Prop-IL-4^o^C	324 ± 1	325 ± 1	323 ± 2	322 ± 2	325 ± 1	
	Prop-SMOF-4^o^C	269 ± 3	269 ± 1	269 ± 1	270 ± 2	272 ± 1	

Values are Mean ± Standard deviation.

**Fig 1:**
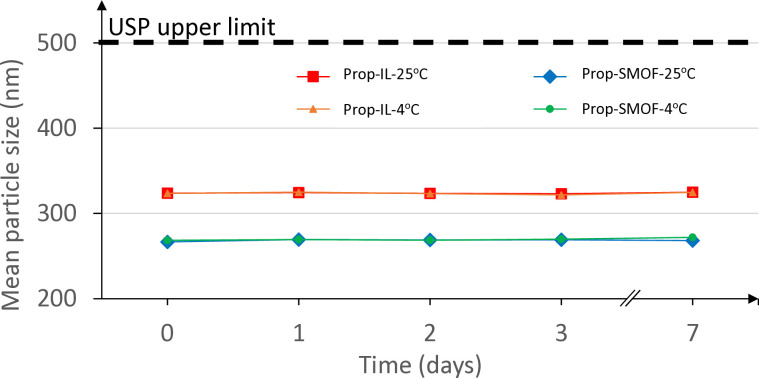
Stability assessment of the average particle size of 12 propofol formulations in INTRALIPID 20% or SMOFlipid 20%. Prop = propofol, IL = Intralipid, SMOF = SMOFlipid.

Particle sizes of the individual formulations remained consistent over time. The stability of mean particle sizes is illustrated in Fig 1, with a maximum variation of 3 nm between the initial mean particle size and the mean particle size at the end of the stability study (after 7 days). This level of variation, approximately 1%, is negligible and likely due to instrumental fluctuations. The average particle size of all formulations remains well below the USP size limit [15].

It is noted that the mean particle sizes of propofol-Intralipid 20% formulations are slightly larger compared to those of propofol-SMOFlipid 20% formulations. Particle sizes for SMOFlipid 20% formulations are around 270 nm, while those for Intralipid 20% formulations are approximately 325 nm. This difference is attributable to the smaller mean particle size of SMOFlipid 20% even before propofol addition. Our formulations also exhibit slightly larger mean particle sizes compared to DIPRIVAN.

Appendix 1 in S1 File displays the size distributions detected using the Beckman Coulter device. The size distributions of our preparations closely match those of the lipid emulsions used prior to propofol addition. Notably, SMOFlipid samples show two distinct size populations, whereas Intralipid samples exhibit only one. Additionally, the particle size distributions remained consistent for at least 7 days.

Moreover, the USP < 729> [15] imposes a limit of 0.05% of globule larger than 5 µm (PFAT5) which we confirmed by detecting no particle larger than 5 µm in any lipid-propofol preparations for at least 7 days by DLS. These results are shown in S1 Table (S1 File).

#### HPLC method selection and validation.

The optimized method detailed in Table 1 was selected primarily for its specificity to propofol, as it effectively separates the peaks of propofol and its main degradation product, Propofol Related Compound B [18]. Validation results for various criteria are provided in Appendix 2 in S1 File. Acceptability criteria for method-specific parameters, such as specificity, column efficiency (N), tailing factor, and coefficient of variation, were based on the USP chapter concerning propofol emulsions for injection [18]. For additional parameters like linearity, precision, and repeatability of the calibration curve, the limits commonly used at the Platform of Biopharmacy, following ICH-Q2(R1) guidelines for validating high-performance liquid chromatography methods, were adhered to [16].

#### HPLC content measurement of formulations.

**Fig 2:**
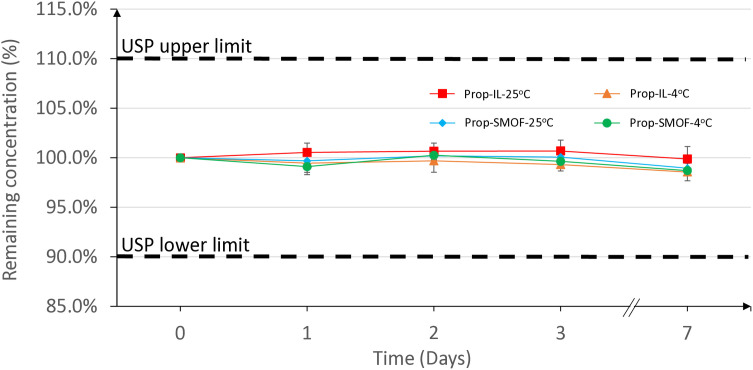
Average remaining concentration of initial content of 12 propofol formulations in INTRALIPID 20% or SMOFlipid 20%. Prop = propofol, IL = Intralipid, SMOF = SMOFlipid; Initial concentrations (mg/ mL): Prop-IL-25^o^C, 9.99 ± 0,10; Prop-SMOF-25^o^C, 9.94 ± 0,04; Prop-IL-4^o^C, 10.13 ± 0.09; Prop-SMOF-25^o^C, 10.05 ± 0,00.

Table 6 indicates that the propofol content in our preparations remained stable throughout the 7-day stability study. Each result represents the average content of 3 vials from the same preparation analyzed in duplicate. The largest decrease from the initial content level was 1.4%. The detected content consistently hovered around 100% of the initial value, suggesting that the variations are largely attributable to measurement method variability, and which is minimal in terms of propofol degradation. The stability of the mean content is clearly illustrated in Fig 2. Given the 10% degradation limit, all our formulations meet the 7-day content stability requirement [18].

**Table 6:** Mean percentage of initial content of 12 propofol formulations in Intralipid 20% (IL) or SMOFlipid 20% (SMOF) (n = 3) kept under different conditions.

Temperature	Sample name	Propofol content (mg/mL)	% of t = 0 propofol content				USP Acceptance criteria [18]
		t = 0	24h	48h	72h	7 days	
25^o^C	Prop-IL-25^o^C	9.99 ± 0,10	100.5% ± 1.0%	100.7% ± 0.8%	100.7% ± 1.1%	99.9% ± 1.3%	[90.0%–110.0%]
	Prop-SMOF-25^o^C	9.94 ± 0,04	99.7% ± 0.8%	100.2% ± 0.6%	100.1% ± 0.9%	98.9% ± 0.6%	
4^o^C	Prop-IL-4^o^C	10.13 ± 0.09	99.4% ± 1.2%	99.7% ± 1.1%	99.3% ± 0.6%	98.6% ± 0.9%	
	Prop-SMOF-4^o^C	10.05 ± 0,00	99.1% ± 0.6%	100.3% ± 0.5%	99.6% ± 0.4%	98.7% ± 0.2%	

Values are Mean ± Standard deviation.

#### pH measurement of preparations.

Table 7 displays the pH values of the marketed products, which will serve as a reference for evaluating the pH of our formulations during the stability study.

**Table 7:** Experimental pH of marketed reference products (n = 1).

Sample	pH
Intralipid 20%	7.62
SMOFlipid 20%	7.30
DIPRIVAN	7.48

**Fig 3:**
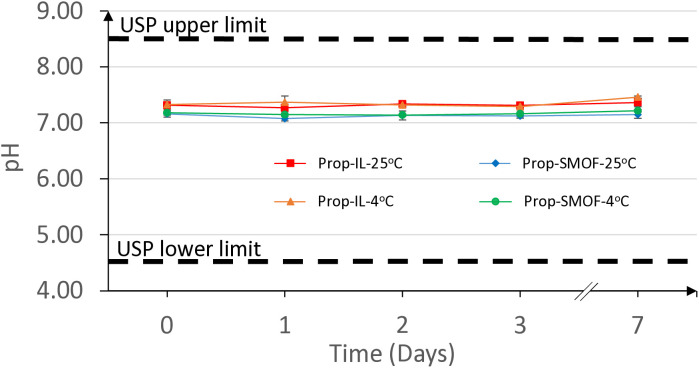
Average pH of 12 propofol formulations in INTRALIPID 20% or SMOFlipid 20% (n = 3). Prop = propofol, IL = Intralipid, SMOF = SMOFlipid.

As detailed in Table 8, the pH of all preparations remained between 7 and 7.5 throughout the entire stability study, with negligible variations. According to USP standards for the pH of injectable propofol preparations, all our formulations are compliant for a minimum duration of 7 days. The stability of the pH is clearly demonstrated in Fig 3.

**Table 8:** Mean pH of 12 propofol formulations in Intralipid 20% (IL) or SMOFlipid 20% (SMOF) (n = 3) kept under different temperature conditions.

Temperature	Sample name	Mean pH					USP Acceptance criteria [18]
		t = 0	24h	48h	72h	7 hours	
25^o^C	Prop-IL-25^o^C	7.31 ± 0.05	7.27 ± 0.07	7.34 ± 0.04	7.31 ± 0.05	7.36 ± 0.07	4.5–8.5
	Prop-SMOF-25^o^C	7.16 ± 0.06	7.08 ± 0.04	7.13 ± 0.08	7.12 ± 0.04	7.15 ± 0.07	
4^o^C	Prop-IL-4^o^C	7.33 ± 0.08	7.37 ± 0.11	7.32 ± 0.04	7.29 ± 0.06	7.46 ± 0.02	
	Prop-SMOF-4^o^C	7.18 ± 0.04	7.15 ± 0.02	7.14 ± 0.02	7.16 ± 0.03	7.22 ± 0.02	

Values are mean ± standard deviation.

## Discussion

In the previous sections, we compared our results primarily with USP criteria for propofol emulsion injections. These criteria are relatively flexible, allowing for significant differences between our preparation and commercially available products. For instance, the USP pH range for injectable emulsions is 4.5 to 8.5, which is quite broad. Therefore, it is more relevant to assess whether our formulations match the characteristics of DIPRIVAN, Intralipid 20%, and SMOFlipid 20% before the addition of propofol.

Our study found that the addition of propofol did not affect the mean particle size or particle size distribution of the two lipid emulsions (Intralipid and SMOFlipid) to which it was added (see Appendix 1 in S1 File). This indicates that propofol is well emulsified within the lipid particles and does not destabilize the emulsions. This result aligns with the known properties of propofol, given its small size and high lipophilicity. Cèbe et al. also observed that the addition of propofol to Intralipid did not affect the mean particle size [11]. This contrasts with findings for nystatin, which increased mean particle sizes in Intralipid [10], suggesting that propofol is more suitable for this formulation technique than larger, more hydrophilic molecules.

The presence of two distinct size populations in propofol-SMOFlipid preparations could be attributed to the multiple surfactants in SMOFlipid, unlike Intralipid, which contains a single surfactant [5,6]. The size distribution of SMOFlipid alone (Appendix 1 in S1 File) confirms that these two populations are not a result of adding propofol. Both formulations had larger mean particle sizes compared to DIPRIVAN. This may be due to a higher surfactant-to-lipid ratio in DIPRIVAN, as it contains 10% lipids compared to the higher lipid concentration in our formulations [1,5,6]. Additionally, DIPRIVAN may undergo additional filtration to retain particles below approximately 400 nm, reducing particle size variability (Appendix 1 in S1 File). Despite this, our formulations meet USP standards for mean particle size.

The USP Chapter <729 > criteria also include PFAT5, the percentage of lipid globules larger than 5 microns, which must be less than 0.05% [15]. This study did not measure PFAT5 due to equipment limitations. However, light diffraction results from the LS Coulter indicated no particles larger than 5 microns as well as by dynamic light scattering as shown in S1 Table (S1 File). The absence of large globule by two different spectroscopic methods proves that our preparation method does not create any large globule in the formulations. Cèbe et al. observed large particles with laser diffraction [11], but we did not, suggesting that our formulation process may have led to better emulsion stability. A key difference is our use of gentle manual shaking, which does not impact the uniformity of propofol content (Table 2), whereas more intense shaking methods, like vortexing, can destabilize emulsions and increase PFAT5 values [11,12]. Our method of gentle shaking likely results in a PFAT5 well below 0.05%, given the absence of particles larger than 1 micron in our samples and consistent particle size distributions before and after propofol addition (Appendix 1 in S1 File).

Our preparations of propofol at a concentration of 10 mg/mL, which is a ready-to-use concentration for propofol emulsion, match one of the concentrations available for DIPRIVAN on the market. The stability study confirmed that propofol is well-integrated into the emulsion, as shown in Fig 2.

The pH results are consistent across our formulations. Comparing the pH values from Tables 7 and 8, we find that adding propofol to either Intralipid 20% or SMOFlipid 20% does not significantly alter the pH. Furthermore, the pH levels are comparable to those of DIPRIVAN for both preparations. This is an improvement from Cèbe et al. preparation that needed pH adjustment [11]. The sterilizing method could be the cause of the pH changes; autoclaving a lipid preparation can promote lipid oxidation, which could have an impact on the product pH [19]. We avoid this problem by using sterilizing filtration which does not involve heating the preparations.

The formulation method we used has been validated by two independent research teams, confirming its viability as a backup option for hospital use. This supports the conclusions of Rooimans et al., which contrast with the findings of Cèbe et al. [11,12]. The oily nature of the propofol drug substance at room temperature aids in the sterilization by filtration, as well as the transfer and mixing of the active ingredient within the injectable vial, facilitating the creation of a sterile final product.

This study offers several advancements over previously published work. We have validated a simpler HPLC method compared to the one proposed by Cèbe et al., while still using the principal propofol degradation compound (RCB) to demonstrate the method specificity—a step not taken by Rooimans et al. [11,12].

Additionally, our approach has been tested with multiple types of parenteral nutrition products. Unlike previous studies that examined only Intralipid 20% or SMOFlipid 20% individually, our study evaluates both lipid emulsions in parallel. This demonstrates that our formulation technique is effective across different lipid emulsions.

This advancement provides a robust alternative to commercial propofol. Should there be a shortage of propofol or Intralipid 20%, SMOFlipid 20% can serve as a viable third option.

## Conclusion

The development of a new formulation method for propofol was necessary as a preventive measure in case of a new period of crisis. During this project, a formulation was developed for propofol and a stability study on this formulation was performed. Our preparation method is simple and only requires products that are normally available in hospitals, due to their wide use there [1,20].

Considering the results of uniformity of content, accuracy of preparation method and stability of our preparations, this study is a success. Using this technique, it may be possible to distribute vials containing only pure propofol to hospitals as a back-up in the event of commercial propofol emulsions running out. Reconstitution can then be carried out by a hospital pharmacist using a predefined volume of Intralipid 20% or SMOFlipid 20%, depending on what is available. Bacterial endotoxins and sterility tests should be performed before distributing such vials in hospitals.

## Supporting information

S1 File Particle size distribution and HPLC method validation. (DOCX)

S2 File HPLC, particle size and pH raw data. (XLSX)

## Abbreviations

ACN: Acetonitrile
DLS: Dynamic Light Scattering
HPLC: High Performance Liquid Chromatography
ICH: International Conference of Harmonization
IL: Intralipid
MeOH: Methanol
PIDS: Polarization Intensity Differential Scattering
Prop.: Propofol
SMOF: SMOFlipid
USP: United States Pharmacopeia
