# Supplementary material for: Propofol emulsification in Intralipid and SMOFlipid: A promising alternative in response to future shortages
Source: PLoS One. 2025 Sep 8;20(9):e0331651. doi: 10.1371/journal.pone.0331651 (PMC12416718; doi:10.1371/journal.pone.0331651)
Supplement: S1 File — (DOCX) [file pone.0331651.s001.docx]

**Propofol emulsification in Intralipid and SMOFlipid: a promising alternative in response to future shortages**

**Compounded formulation for propofol in response to shortages**

**Maxime Murphy^1^ (B.Sc.), Mihaela Friciu^1^ (M.Sc.), Valérie Gaëlle Roullin^1^ (Ph.D.), Grégoire Leclair^1*^ (Ph.D.)**

*^1^Plateforme de biopharmacie, Université de Montréal, 2940 Chem. de la Polytechnique, Montréal, QC H3T 1J4, Qc, Canada*

* Corresponding author

[gregoire.leclair@umontreal.ca](mailto:gregoire.leclair@umontreal.ca)

***Supporting information file #1***

***Particle size distribution and HPLC method validation***

Table of content

[I. Appendix 1: Particle size distribution 3](#_Toc200710835)

[II. Appendix 2: HPLC method validation 6](#_Toc200710836)

[A. HPLC-UV method– Variability 6](#_Toc200710837)

[Intra-day variability 6](#_Toc200710838)

[Inter-day variability 6](#_Toc200710839)

[B. HPLC-UV method – Linearity 7](#_Toc200710840)

[C. HPLC-UV method – Accuracy 9](#_Toc200710841)

[D. HPLC-UV method – Specificity and efficiency of the method 9](#_Toc200710842)

[**Figure S 1: Particle size distribution of marketed lipid emulsions and propofol preparations at the start and end of the stability study*** A: Marked lipid emulsion and Diprivan particle size distribution. B: Propofol compounded formulations particle size distribution kept at 4^o^C and 25^o^C at t=0 and 7 days after the beginning of the stability study. 4](#_Toc200710948)

[**Figure** **S** **2**: **Pictures of a Intralipid-propofol preparation kept at ambient temperature at the beginning of the stability study (t=0) and at the end of it (t=7 days)**.** 5](#_Toc200710949)

[**Figure S 3 :** **Calibration curves for the same propofol samples diluted in 1:1 ACN:MeOH on three consecutive days.** A: Calibration curve injected on day 1. B: Calibration curve injected on day 2. C: Calibration curve injected on day 3. 8](#_Toc200710950)

[**Figure S 4: Chromatograms of propofol, Propofol Related Compound B (RCB) and the mixture of these two products obtained with the validated HPLC method.** 10](#_Toc200710951)

[**Table S 1: Assessment of particle larger than 5 µm in propofol preparations by DLS at the start and end of the stability study** 5](#_Toc200710857)

[**Table S 2: Identification and concentration of samples used for method variability and calibration curve tests.** 6](#_Toc200710858)

[**Table S 3: Peak areas of propofol in HPLC at four points in time on the same day** 6](#_Toc200710859)

[**Table S 4: Mean peak area of propofol in HPLC at 24-hour intervals.** 6](#_Toc200710860)

[**Table S 5: Calibration curve parameter values for the same samples injected on 3 different days.** 7](#_Toc200710861)

[**Table S 6: Accuracy of the different calibration curves as a function of the regressed concentration value and the experimental value obtained.** 9](#_Toc200710862)

[**Table S 7: Specificity and efficiency parameters required to validate the HPLC method according to USP criteria.** 10](#_Toc200710863)

# Appendix 1: Particle size distribution

A

**Intralipid 20% alone: DIPRIVAN:**

| 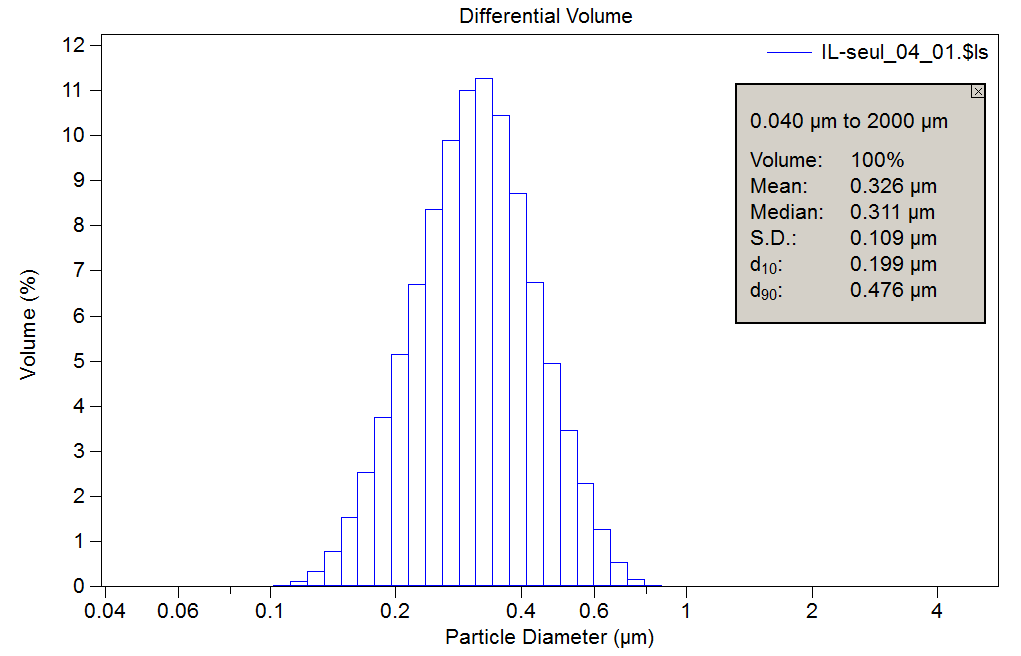  **SMOFlipid 20% alone:** | 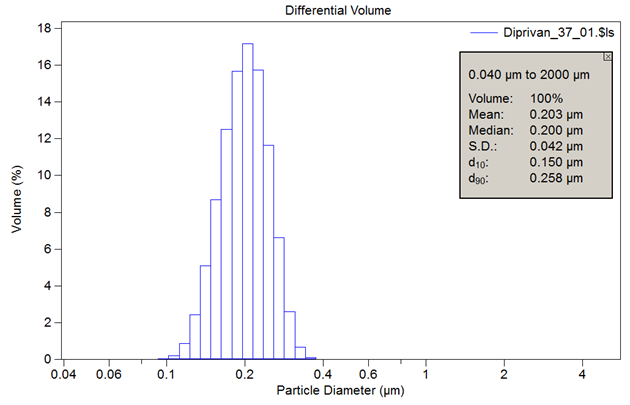 |
| --- | --- |
| 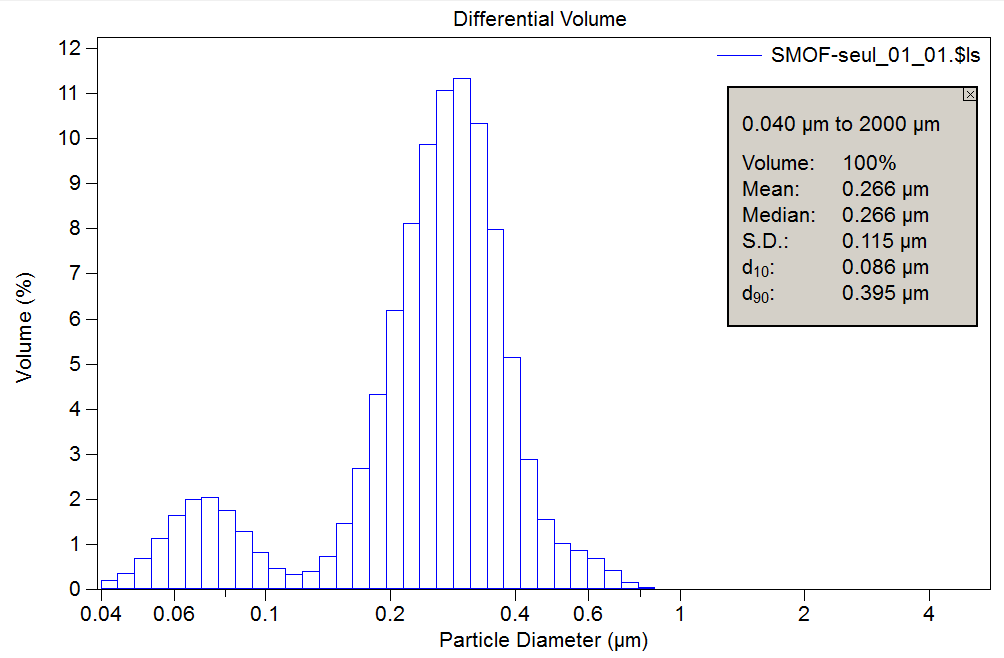 |  |

B

**t=0 t= 7 days**

**Propofol-Intralipid at 25^o^C:**

| 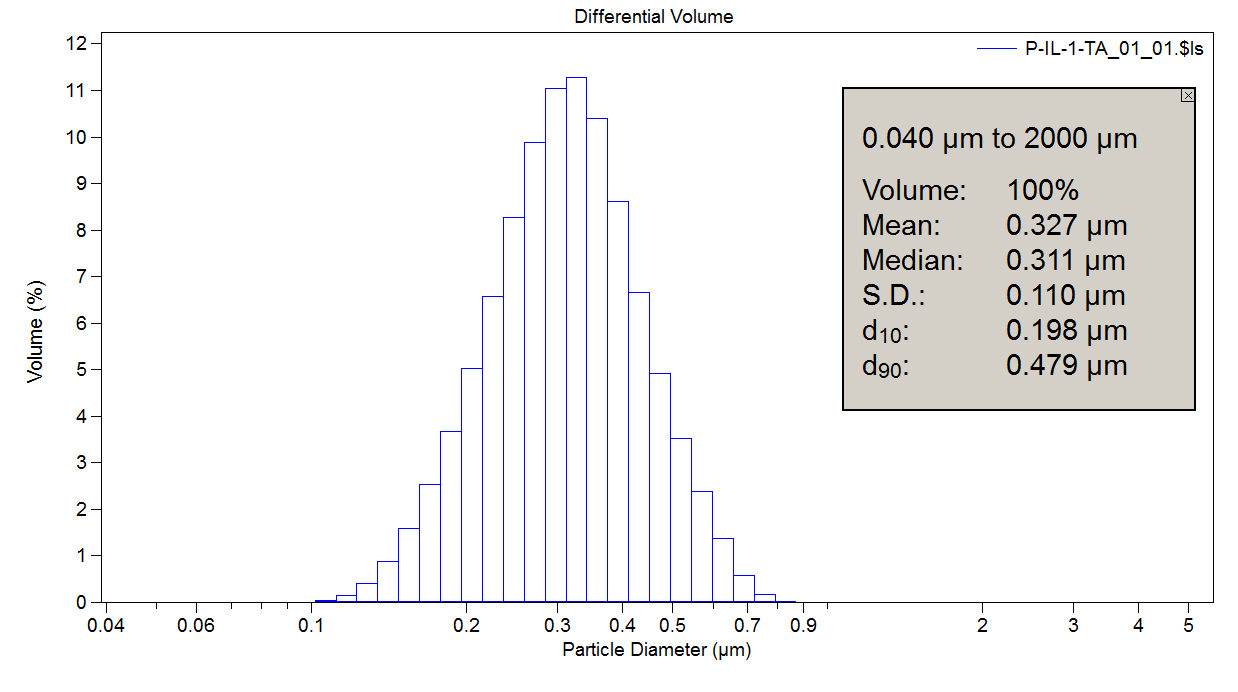 | 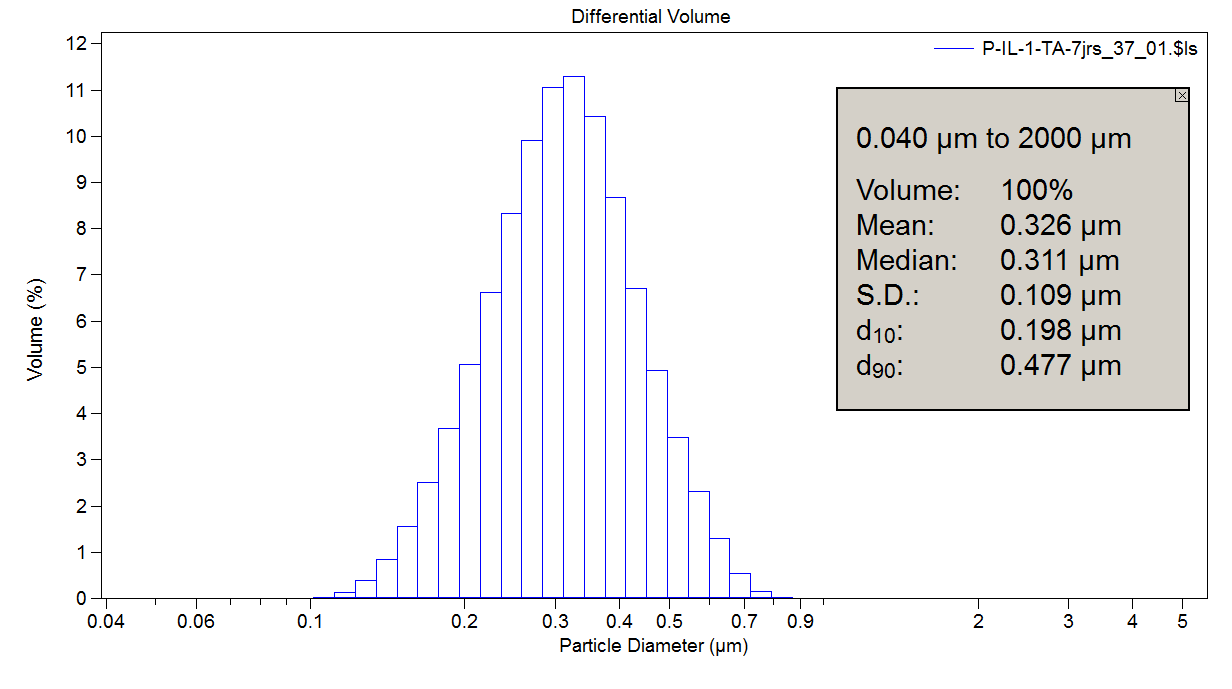 |
| --- | --- |

**t=0 t= 7 days**

**Propofol-Intralipid 20% at 4^o^C:**

| 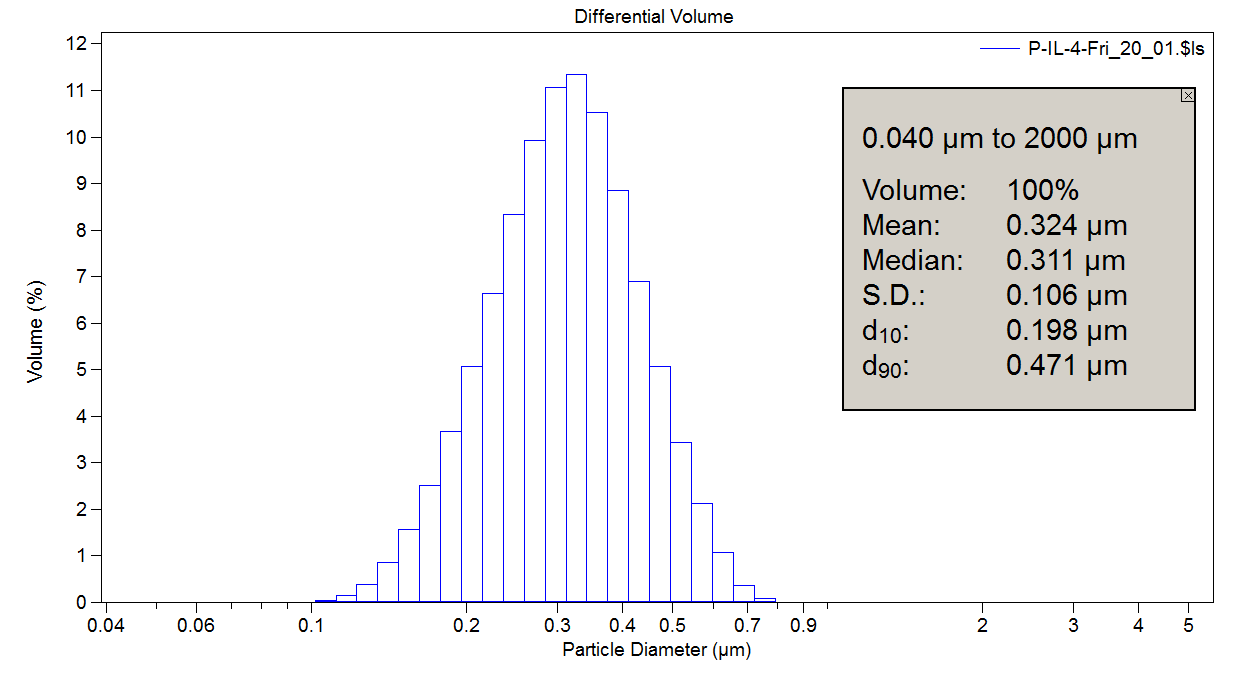 | 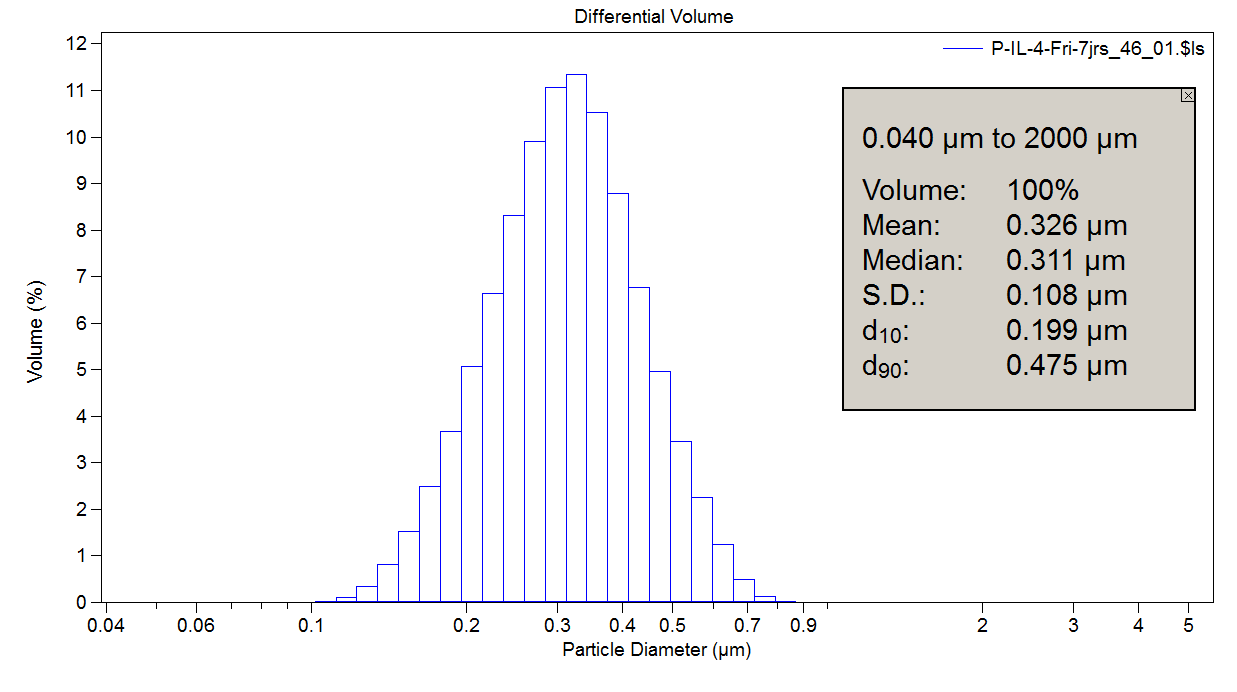 |
| --- | --- |

**Propofol-SMOFlipid 20% at 25^o^C:**

| 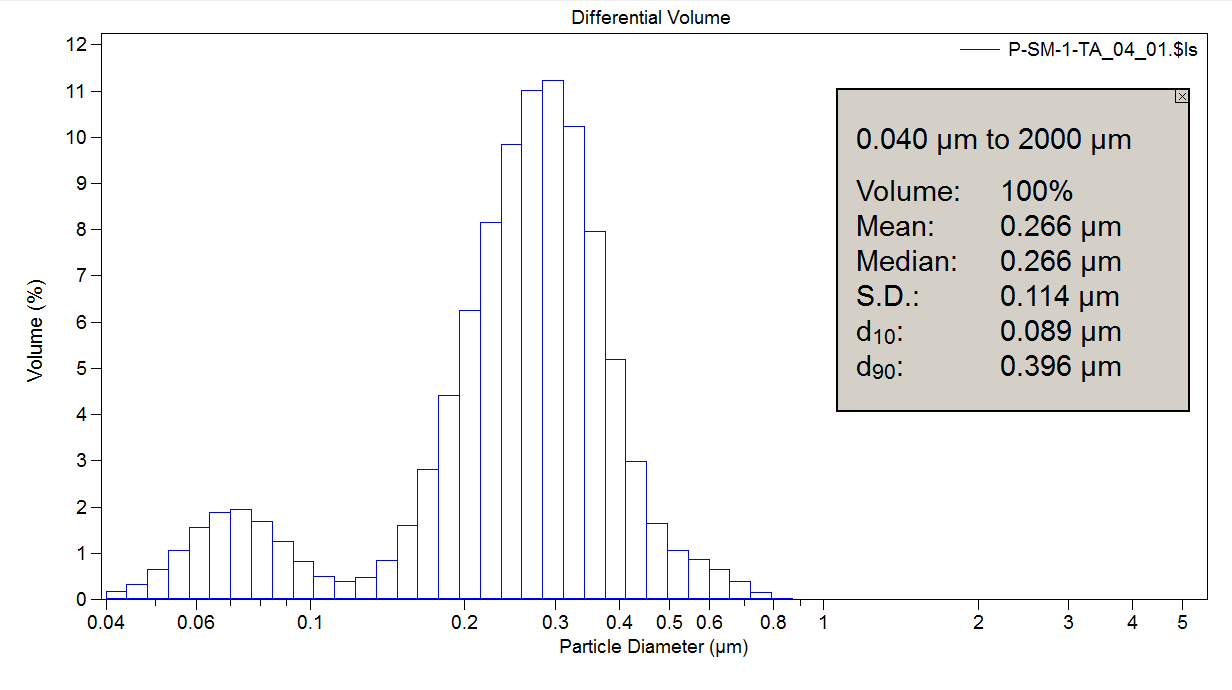 | 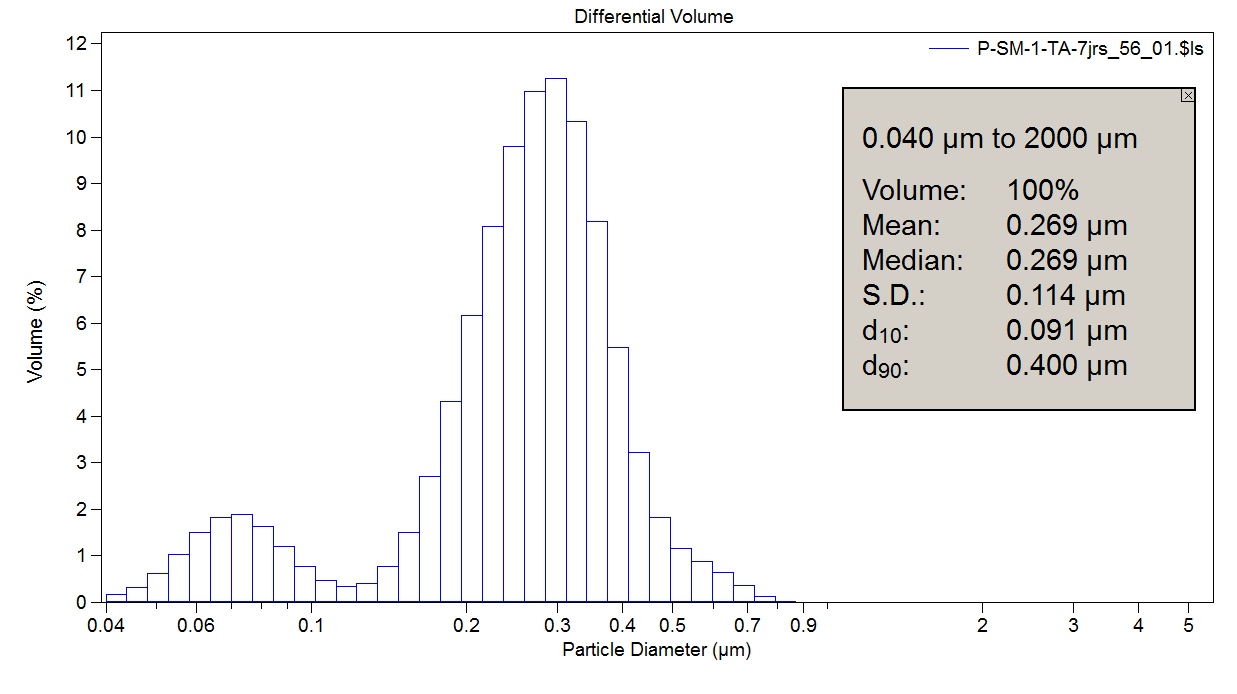 |
| --- | --- |

**Propofol-SMOFlipid 20% at 4^o^C:**

| 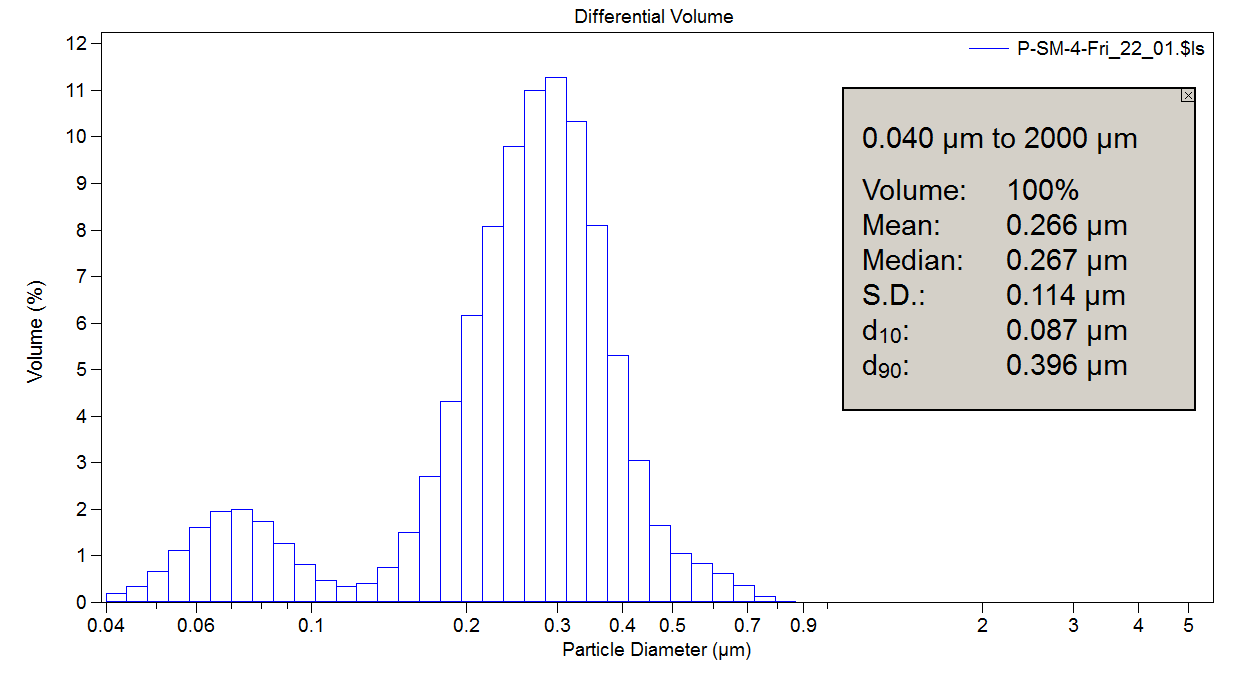 | 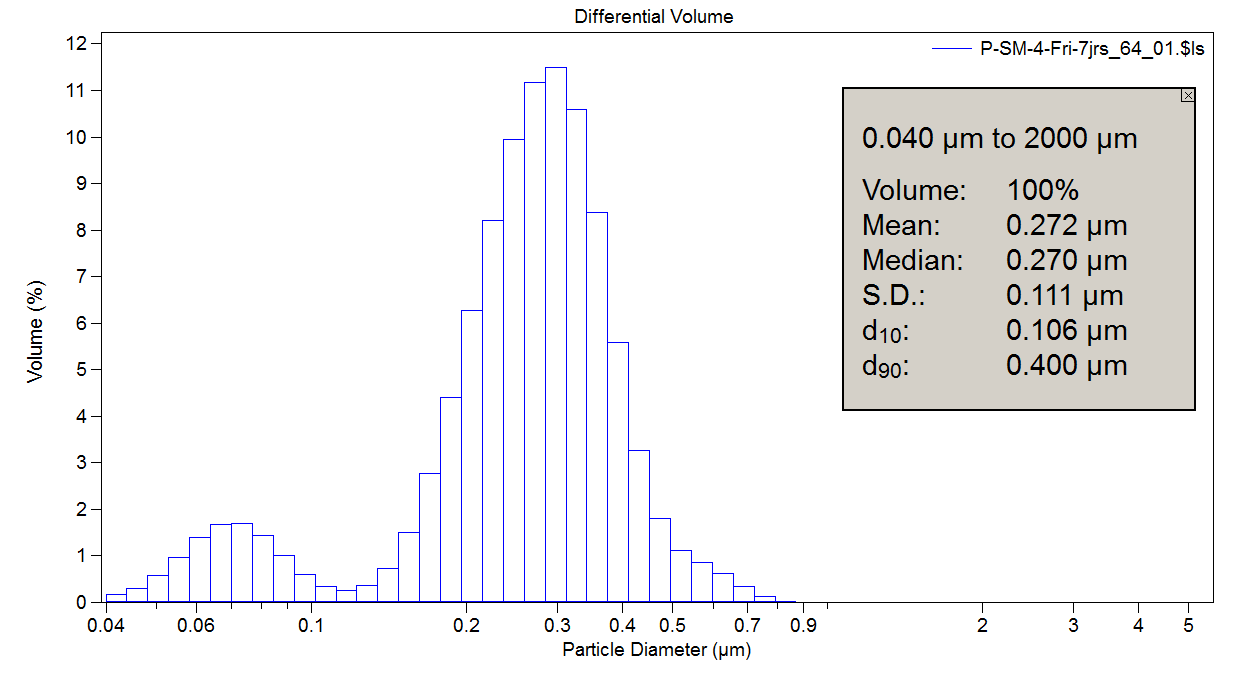 |
| --- | --- |

**Figure S 1: Particle size distribution of marketed lipid emulsions and propofol preparations at the start and end of the stability study*** A: Marked lipid emulsion and Diprivan particle size distribution. B: Propofol compounded formulations particle size distribution kept at 4^o^C and 25^o^C at t=0 and 7 days after the beginning of the stability study.

*Note that the size distributions of only a few samples are shown here. However, the distributions were so identical to each other, that it can be assumed that all the size distributions in Intralipid 20% and SMOFlipid 20% are identical to those shown in the figure above.

**Table S 1: Assessment of particle larger than 5 µm in propofol preparations by DLS at the start and end of the stability study**

| Sample | Biggest particle detected by DLS (µm) | | Number of particle bigger than 5 µm | |
| --- | --- | --- | --- | --- |
|  | t=0 | t=7 | t=0 | t=7 |
| Prop-IL-25^o^C | 1.169 | 1.650 | 0 | 0 |
| Prop-SMOF-25^o^C | 0.926 | 1.204 | 0 | 0 |
| Prop-IL-4^o^C | 2.165 | 1.135 | 0 | 0 |
| Prop-SMOF-4^o^C | 0.966 | 0.978 | 0 | 0 |

The raw data for particle size determination by Coulter is available in the Excel document titled S2 File: HPLC, Particle Size and pH Raw Data. While a summary table has been prepared, due to the large volume of raw data for this part of the project (over 200 Excel files), the original data files exported directly from the Coulter and DLS software will be made available upon request.

| **t=0** | **t= 7 days** |
| --- | --- |
| 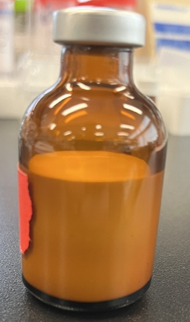 | 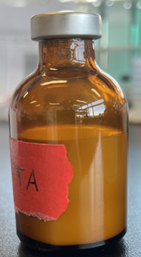 |

**Figure** **S** **2**: **Pictures of an Intralipid-propofol preparation kept at ambient temperature at the beginning of the stability study (t=0) and at the end of it (t=7 days)**.**

**Every preparation had the same appearance during the entirety of the stability study, no matter the temperature or the lipid used.

# Appendix 2: HPLC method validation

## HPLC-UV method– Variability

For method variability results, 5 samples of propofol diluted in the solvent used for sample extraction (1:1 ACN:MeOH) were injected four times a day for a period of 3 days. These samples corresponded to a serial dilution of a stock solution containing 101.62 mg of diluted propofol to obtain a final volume of 100 mL, followed by a further dilution in the proportions given in Table IX. The same samples were injected each day. The same samples were injected each day, enabling us to create a propofol calibration curve for our stability study.

**Table S 2: Identification and concentration of samples used for method variability and calibration curve tests.**

| Name of the sample | Volume of stock solution (µL) | Concentration of stock solution (mg/mL) | Diluent volume (µL) | Actual sample concentration (µg/mL) |
| --- | --- | --- | --- | --- |
| 80% | 80 | 1.02 | 920 | 81.30 |
| 90% | 90 |  | 910 | 91.46 |
| 100% | 100 |  | 900 | 101.62 |
| 110% | 110 |  | 890 | 111.78 |
| 120% | 120 |  | 880 | 121.94 |

### Intra-day variability

**Table S 3: Peak areas of propofol in HPLC at four points in time on the same day**

| Sample | Peak 1 area | Peak 2 area | Peak 3 area | Peak 4 area | Mean area | Standard deviation | RSD (%) | Acceptation |
| --- | --- | --- | --- | --- | --- | --- | --- | --- |
| 80% | 730 414 | 729 756 | 729 749 | 732962 | 730 720 | 1527 | 0.2% | RSD ≤ 2% |
| 90% | 826 093 | 824 065 | 825 605 | 831836 | 826 900 | 3402 | 0.4% | RSD ≤ 2% |
| 100% | 914 595 | 910 470 | 911 222 | 920857 | 914 286 | 4734 | 0.5% | RSD ≤ 2% |
| 110% | 1 009 843 | 1 008 544 | 1 007 885 | 1014132 | 1 010 101 | 2808 | 0.3% | RSD ≤ 2% |
| 120% | 1 099 473 | 1 097 529 | 1 098 000 | 1108094 | 1 100 774 | 4950 | 0.4% | RSD ≤ 2% |

### Inter-day variability

**Table S 4: Mean peak area of propofol in HPLC at 24-hour intervals.**

| Sample | Mean peak area at t=0 (n=4) | Mean peak area at 24 h (n=4) | Mean peak area at 48 h (n=4) | Mean area | Standard deviation | RSD (%) | Acceptation |
| --- | --- | --- | --- | --- | --- | --- | --- |
| 80% | 730 720 | 735 981 | 744 294 | 736999 | 6844 | 0.9% | RSD ≤ 3% |
| 90% | 826 900 | 833 267 | 846 355 | 835507 | 9919 | 1.2% | RSD ≤ 3% |
| 100% | 914 286 | 927 485 | 939 993 | 927255 | 12855 | 1.4% | RSD ≤ 3% |
| 110% | 1 010 101 | 1 016 645 | 1 028 001 | 1018249 | 9057 | 0.9% | RSD ≤ 3% |
| 120% | 1 100 774 | 1 116 458 | 1 139 334 | 1118855 | 19392 | 1.7% | RSD ≤ 3% |

## HPLC-UV method – Linearity

The linearity of the calibration curve mainly determines how well it determines the concentration of solutions over a predefined concentration range. The extraction of samples during HPLC measurements in the stability study dilutes them, so the concentrations detected by the HPLC area do not correspond to the concentration of the preparation. A dilution factor must therefore be considered to determine the concentration detected as a function of the peak area. This dilution factor can be considered directly in the calibration curve. In fact, the dilution factor is always the same, so it is possible to change the units in the calibration curve to have a content of around 10 mg/mL as a function of area, although the real concentration of the samples used to construct the calibration curve is around 100 µg/mL.

**Table S 5: Calibration curve parameter values for the same samples injected on 3 different days.**

| Parameter | | t=0 | 24 h | 48 h | RSD (%) | Acceptation |
| --- | --- | --- | --- | --- | --- | --- |
| Determination coefficient (R^2^) | | 0.9998 | 0.9997 | 0.9987 |  | ≥ 0.99 |
| Slope | | 90859 | 92928 | 95624 | 3% | CV ≤ 3% |
| Y-Intercept | Value | -6752.6 | -18363 | -32131 | -67% |  |
|  | % of 100% Area | 1% | 2% | 3% |  | ≤ 3% |

A

B

C

**Figure S 3 :** **Calibration curves for the same propofol samples diluted in 1:1 ACN:MeOH on three consecutive days.** A: Calibration curve injected on day 1. B: Calibration curve injected on day 2. C: Calibration curve injected on day 3.

## HPLC-UV method – Accuracy

**Table S 6: Accuracy of the different calibration curves as a function of the regressed concentration value and the experimental value obtained.**

| Sample | Accuracy | | | Mean | Standard deviation | Inter-day RSD (%) | Acceptation |
| --- | --- | --- | --- | --- | --- | --- | --- |
|  | t=0 | 24 h | 48 h |  |  |  |  |
| 80% | 99.84% | 99.85% | 99.88% | 99.86% | 0.02% | 0.02% | 95%-105% |
| 90% | 100.32% | 100.20% | 100.45% | 100.32% | 0.13% | 0.12% | 95%-105% |
| 100% | 99.75% | 100.16% | 100.04% | 99.98% | 0.21% | 0.21% | 95%-105% |
| 110% | 100.12% | 99.64% | 99.18% | 99.65% | 0.47% | 0.47% | 95%-105% |
| 120% | 99.96% | 100.14% | 100.46% | 100.19% | 0.25% | 0.25% | 95%-105% |

## HPLC-UV method – Specificity and efficiency of the method

The specificity of the HPLC method is important if it is to detect the degradation of propofol into its degradation products. To do this, the peak corresponding to propofol must be completely separated from the peaks corresponding to the degradation products of this active ingredient. In our case, the USP chapter corresponding to propofol lipid emulsions for injection mentions that the only degradation product that needs to be well separated from propofol to validate the method is Propofol Related Compound B (RCB). This compound is very similar to propofol, so it is difficult to separate from it. In our case, we purchased a vial containing RCB that was supposedly 97.01% pure. However, as can be seen in *Figure 5*, the sample corresponding to RCB alone shows several peaks with different retention times. This could be because the RCB itself has degraded into other products, or because the sample was not pure. However, this has no impact on the validation of the method, since the sample contains at least a certain amount of RCB, and all degradation peaks are well separated from the main peak of propofol. Our method is therefore specific for propofol. Our method is also acceptable from the point of view of the column's tailing factor and number of theoretical plateaus. (15)

The raw data for HPLC and pH results are available in the Excel document titled S2 File: HPLC, Particle Size and pH Raw Data.

**Table S 7: Specificity and efficiency parameters required to validate the HPLC method according to USP criteria.**

| Product | Retention time (min) | Area | Tailing | | Resolution | | Number of theoretical plateaus (N) | | Peak width (min) |
| --- | --- | --- | --- | --- | --- | --- | --- | --- | --- |
|  |  |  | Value | Acceptation | Value | Acceptation | Value | Acceptation |  |
| Propofol | 12.581 | 483419 | 0.963 | ≤ 1.5 | 2.873 | ≥ 2.5 | 1850 | >1000 | 1.17 |
| RCB (1) | 7.872 | 117703 | 0.969 |  | 3.2 |  |  |  |  |
| RCB (2) | 10.868 | 35269 | 0.939 |  | 3.236 |  |  |  |  |


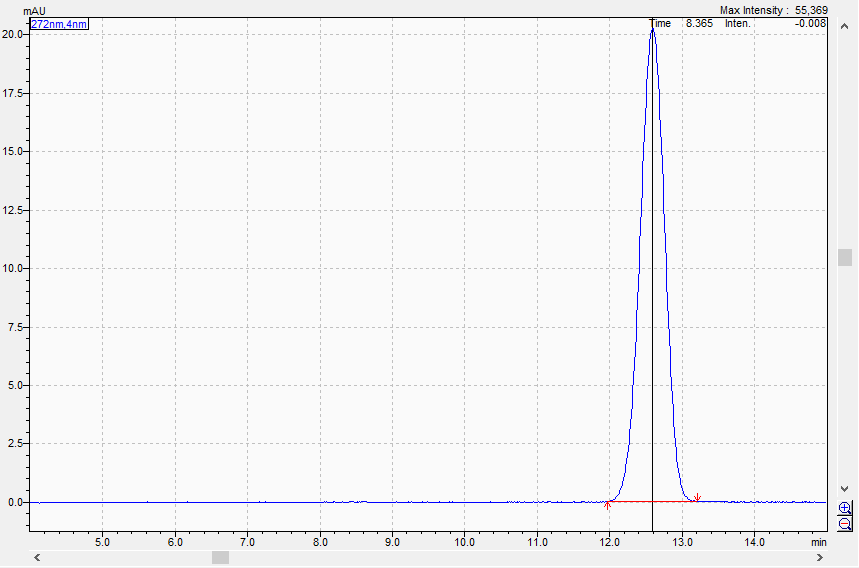
***
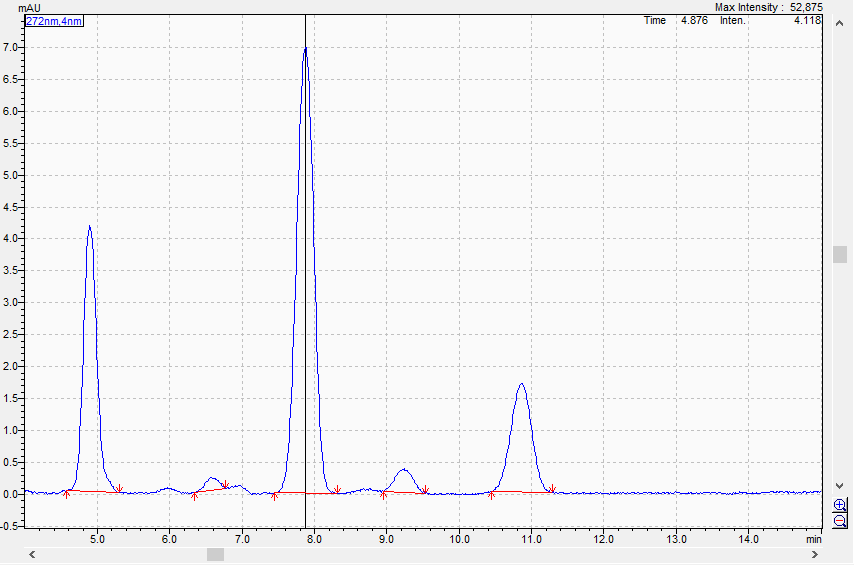
***


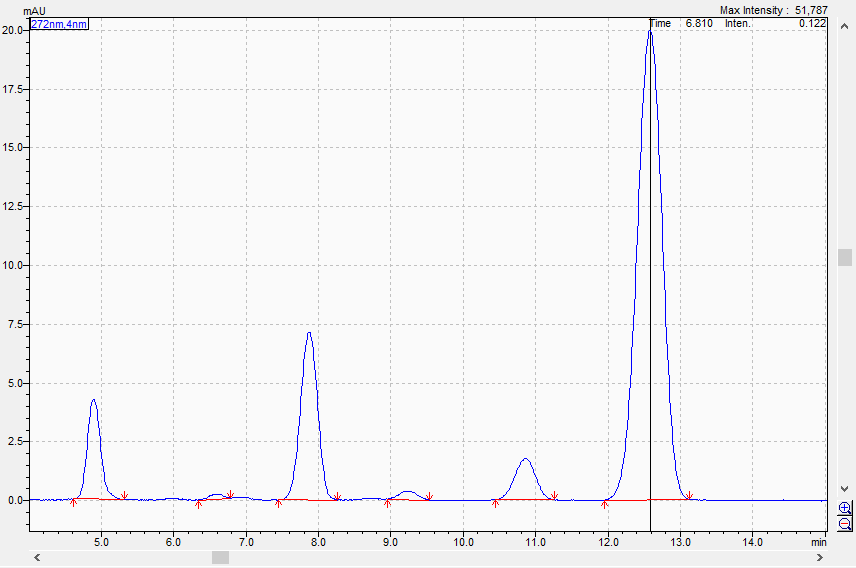
 ***Propofol alone RCB alone***

**Propofol + RCB**

**Figure S 4: Chromatograms of propofol, Propofol Related Compound B (RCB) and the mixture of these two products obtained with the validated HPLC method.**
