# Supplementary material for: Propofol emulsification in Intralipid and SMOFlipid: A promising alternative in response to future shortages
Source: PLoS One. 2025 Sep 8;20(9):e0331651. doi: 10.1371/journal.pone.0331651 (PMC12416718; doi:10.1371/journal.pone.0331651)
Supplement: Attachment — Submitted filename: Response to Reviewers.docx [file pone.0331651.s004.docx]

**Rebuttal letter:**

Dear Dr Giordano,

We would like to thank you and the reviewers for your time, careful reading, and insightful comments on our manuscript titled ‘Propofol emulsification in Intralipid and SMOFlipid: a promising alternative in response to future shortages’ (Manuscript ID: PONE-D-25-20660). We greatly appreciate the constructive feedback, which has helped us improve the clarity, scientific rigor, and overall quality of the manuscript.

We believe we have positively addressed all comments related to Journal Requirements. Financial disclosure was updated in the Cover Letter. Funding Information was also updated. Both now match. All funding information was removed from the manuscript.

You will also find below our detailed responses to each point of the Comments to the Authors. All changes made to the manuscript in response to the reviewers’ suggestions have been clearly marked in the revised version.

We hope that the revised manuscript meets the expectations for publication in Plos One, and we remain at your disposal for any further clarification.

Sincerely,

Dr Grégoire Leclair, On behalf of all authors.

Faculty of pharmacy, University of Montreal

**Comments to the Authors**

We thank the reviewers for their time and effort. Their valuable comments have undoubtedly helped us improve the quality and clarity of this manuscript.

**Reviewer #1**: 1. The manuscript proposes that Lipocalin-2 (Lcn2) deficiency exacerbates ileitis via effects on microbiota and mucosal immunity. While the rationale is interesting and relevant, the hypothesis needs to be better structured and clearly articulated in the introduction. A concise paragraph that lays out the working model at the end of the introduction would significantly enhance readability.

2. The 16S rRNA sequencing results are central to the study. However, key details on sequencing depth, controls, and statistical robustness are missing. For instance, rarefaction curves or measures of sequencing coverage (e.g., Good’s coverage) are not discussed. Additionally, the choice of alpha- and beta-diversity metrics needs justification. Include more robust statistical validation for LEfSe results.

3. Some bar plots lack error bars (e.g., in microbiota composition figures). Also, legends should include sample sizes (n) and significance indicators.

4. While generally readable, the manuscript contains grammatical errors and awkward phrasing (e.g., "Lcn2 knockout mice are more prone to intestinal inflammation due to defective immune"). Consider professional English editing

5. Some abbreviations (e.g., FMT, DSS, LP) are used before being defined.

However, the conclusions are not fully supported by the current data presentation and lack mechanistic depth. With significant revisions, especially in clarifying experimental design, statistical analysis, and integrating functional insight, the manuscript could become a valuable contribution to the field.

We thank the reviewer for agreeing to revise our manuscript. However, the comments provided appear to pertain to a different subject than the one addressed in our work. As such, we believe these comments fall outside the scope of our manuscript and have not incorporated them into our revision.

**Reviewer #2:** In the provided manuscript, it has been suggested that it may be possible to distribute vials containing only pure propofol to hospitals as a back-up in the event of commercial propofol emulsions running out. Reconstitution can then be carried out by a hospital pharmacist using a predefined volume of Intralipid 20% or SMOFlipid 20%, depending on what is available. Bacterial endotoxins and sterility tests should be performed before distributing such vials in hospitals which in my view is a critical challenge and risk for the overall usability of this alternative product. It would be strongly recommended to evaluate and demonstrate the shelf life of reconstituted propofol emulsions with long term chemical stability as well as for sterility against microbial growth and contamination.

We thank the reviewer for this thoughtful comment regarding the sterility and stability of the proposed reconstituted propofol emulsions.

We would like to clarify that the approach we describe is intended strictly as a *backup solution*, to be employed only in the event of a critical shortage of commercially available propofol emulsions. Under such exceptional circumstances, the formulation would be prepared by a hospital pharmacist and administered shortly thereafter—within minutes to a few hours—thus minimizing concerns related to microbial contamination or endotoxin formation.

Importantly, the vials distributed to hospitals would contain only sterile, pure propofol, a substance with a well-established chemical stability profile, similar to reference standards obtained from certified suppliers (e.g., Toronto Research Chemicals). In cases where the vials cannot be used promptly, storage at −20 °C would further ensure both chemical stability and sterility, as propofol solidifies under these conditions, effectively limiting microbial growth and endotoxin development.

Regarding the reconstituted emulsions, we have already demonstrated chemical stability for up to 7 days. However, we emphasize that in the scenario we envision, the emulsion would be prepared and administered on the same day, further reducing any practical relevance of long-term stability or sterility concerns.

Given the emergency-use context, the immediate-use timeline, and the existing data on stability, we believe that additional long-term sterility and endotoxin studies, while certainly valuable under other circumstances, are not strictly necessary for the specific purpose and usage scenario we are addressing.

The number of samples (n) tested in the study is too low to carry out any meaningful statistical analysis across multiple experiments. it would be strongly recommended to add more samples to the experimental studies and carry out statistical analysis to further support the reliability and reproducibility of the data.

We appreciate the reviewer’s suggestion regarding the use of a larger number of samples and more extensive statistical analysis.

However, the number of replicates used in our study (n = 3 per condition, across 4 conditions) is in line with current regulatory guidelines for stability studies of compounded formulations. This approach reflects established practice and is considered sufficient by agencies such as the FDA and EMA to assess the reproducibility and reliability of results in this context.

Our results showed consistent and reproducible outcomes across replicates, which supports the robustness of the formulation under the tested conditions. While larger sample sizes and formal statistical tests can be valuable in broader pharmacological or clinical studies, they are not typically required—or expected—for stability testing in pharmaceutical compounding, where the focus is on observing trends and verifying reproducibility under defined storage conditions.

We hope this clarification addresses the reviewer’s concern.

**Reviewer #3:** Methodological Clarifications and Recommendations for Improvement

The manuscript titled "Propofol emulsification in Intralipid and SMOFlipid: a promising alternative in response to future shortages" presents an interesting and timely approach. However, to enhance scientific rigor, reproducibility, and alignment with best practices, the following issues must be addressed prior to consideration for publication:

1. Preparation Methodology:

The authors mention that emulsification was achieved through “gentle mixing,” but no further methodological details regarding time etc. are provided. It is essential to specify whether shaking was performed manually or using a mechanical device (e.g., vortex mixer or orbital shaker). If mechanical mixing was used, details such as equipment type, angle (if applicable), speed (rpm), and duration must be included. This information is critical for ensuring reproducibility and for interpreting the consistency and quality of the emulsion.

We thank the reviewer for highlighting the importance of methodological transparency.

In response, we have added two detailed paragraphs to the section “Propofol Compounded Formulation Preparation” (beginning at line 119 of the revised manuscript). These additions describe the manual shaking process used to achieve emulsification, including all relevant parameters such as duration, intensity, and technique.

We believe this additional information now ensures full reproducibility and addresses the reviewer’s concern regarding preparation consistency and emulsion quality.

2. Drug Extraction for HPLC Analysis:

While the extraction solvent is mentioned, the manuscript does not provide sufficient detail on the extraction procedure. The authors should clarify:

The number of serial extraction steps performed to ensure complete recovery of Propofol; The volume of solvent used per step; Whether the extracts were pooled or analyzed individually; Data supporting extraction efficiency, if any.

These details are important for the reliability of quantitative results and should conform to ICH Q2(R1) requirements for method validation.

We appreciate the reviewer’s attention to the extraction methodology, which is indeed crucial for ensuring the reliability and reproducibility of quantitative results.

In response, we have added the requested details in the two **“Propofol Extraction”** subsections, starting at **line 248** of the revised manuscript. Specifically, we now clarify that:

- A single extraction step was sufficient to achieve near-complete recovery (~100 ± 0.3%) for Propofol in SMOFlipid preparations.
- The extraction was performed using **9.9 mL** of solvent added to **100 µL** of propofol emulsion, for a total volume of **10 mL**.
- Extracts were **analyzed individually**, not pooled.
- Recovery data are provided, demonstrating that the method yields consistent and quantitative extraction of propofol, in line with expectations for analytical accuracy.

We believe these additions bring the procedure into alignment with best practices and the expectations outlined in ICH Q2(R1) for analytical method validation.

3. HPLC Method Parameters and System Suitability:

The manuscript outlines the main chromatographic conditions; however, key validation parameters required by USP and ICH guidelines are missing. The authors should include:

- System suitability data, including tailing factor, resolution (if applicable), theoretical plate count, and %RSD for retention time and peak area
- Method validation data, such as linearity, precision, accuracy, LOD/LOQ, only in table form (authors may be planning to submit a separate manuscript for HPLC -Analytical Method Development and Validation).

We thank the reviewer for raising this important point regarding method validation in accordance with USP and ICH guidelines.

A complete validation of the HPLC method—including system suitability parameters (e.g., tailing factor, theoretical plate count, %RSD for retention time and peak area) as well as method validation data (linearity, precision, accuracy, specificity)—has been provided in the **Supporting Information** of the manuscript.

We made the choice to include this information as supplementary material to maintain the focus of the main text on the practical and pharmaceutical aspects of the study, which we believe are of primary interest to medical professionals and compounding pharmacists. Nonetheless, we agree that these validation details are critical for ensuring analytical reliability and transparency, which is why they are fully documented and accessible in the supporting files.

4. Visual Representation:

To enhance clarity and facilitate reproducibility, the manuscript would benefit from the inclusion of photographs or schematic diagrams of:

- The emulsion preparation process
- Visual appearance of stability samples at various time points
- Pictures of Output of results generated by equipment, if available.

These additions can aid in interpretation and provide qualitative support to the analytical findings.

We thank the reviewer for this constructive suggestion.

While we initially questioned the added value of including visuals for a process as simple as combining propofol with a lipid emulsion in a vial, we agree that visual representation can help support clarity and reproducibility. As such, we have included **photographs of an emulsion preparations at Day 0 and Day 7** in the **Supporting Information** to illustrate the visual appearance over time.

Regarding analytical output visuals, we would like to point out that we have already included:

- A representative **particle size distribution graph** from the LS Coulter (**Figure S1**)
- A **chromatogram from the specificity validation** of the HPLC method (**Figure S3**)

These visual elements, already present in the Supporting Information, contribute to the interpretation and qualitative assessment of both formulation stability and analytical performance.

We hope these additions fully address the reviewer’s recommendation.

5. Particle Size Distribution and Zeta Potential:

Given that the study involves emulsified formulations, characterization of physical stability is essential. It is recommended that the authors include:

- Particle size distribution (e.g., mean droplet diameter, polydispersity index) using dynamic light scattering or an equivalent technique
- Zeta potential measurements to assess electrostatic stability (ideally ±30 mV for stable emulsions)
- Stability data for these parameters over time to support conclusions regarding formulation robustness

This is in line with best practices and regulatory expectations (Health Canada /EMA/FDA) for injectable emulsions.

We thank the reviewer for highlighting the importance of physical characterization in evaluating emulsion stability.

Particle size distribution data have been included in the **Supporting Information** (**Figure S1 and Table S1**). These were obtained using laser diffraction with an LS Coulter instrument, a technique recognized by USP <729> as a valid and widely accepted method for evaluating mean droplet size in lipid injectable emulsions. While parameters such as polydispersity index (PDI) are informative, they are not required by regulatory agencies (e.g., Health Canada, EMA, FDA) for compounded emulsions.

Our study employed Intralipid and SMOFlipid, both of which are already approved and marketed lipid emulsions that conform to stringent regulatory standards for particle size and stability. Our results demonstrate that the addition of propofol does not significantly alter the particle size distribution of these emulsions, supporting the suitability of the **compounded** formulation for parenteral administration.

Regarding zeta potential, we acknowledge that this parameter can provide useful insights into electrostatic stability. However, our laser diffraction setup does not support this measurement, and such equipment is far less commonly available in hospital or compounding settings. Nonetheless, the consistency of the particle size profiles over the full duration of the stability study strongly supports the physical stability of the emulsions. These data are presented in **Table 5** of the main manuscript and in the Supporting Information (**Figure S1 and Table S1, Appendix 1**).

In summary, while zeta potential could further support our findings, we believe that the presented data are in line with current regulatory expectations for injectable emulsions and adequately support the physical robustness of the formulations.

6. Check for spelling -International Conference on Harmonisation-ICH

Thank you for pointing this out. The spelling of International Conference on Harmonisation (ICH) has been reviewed and corrected throughout the manuscript.

The study addresses an important formulation challenge; however, the above methodological gaps need to be rectified to meet the standards of scientific rigor and regulatory compliance. I recommend revision of the manuscript incorporating these detailed improvements.

We sincerely thank the reviewer for recognizing the relevance of our work in addressing an important formulation challenge. We also appreciate the detailed and constructive feedback aimed at strengthening the methodological rigor and regulatory alignment of our study.

In response, we have carefully revised the manuscript to address each of the points raised. We believe these changes have significantly improved the clarity, reproducibility, and scientific robustness of the work, and we hope the revised version meets the standards expected for publication.
