# Supplementary material for: Propofol emulsification in Intralipid and SMOFlipid: A promising alternative in response to future shortages
Source: PLoS One. 2025 Sep 8;20(9):e0331651. doi: 10.1371/journal.pone.0331651 (PMC12416718; doi:10.1371/journal.pone.0331651)
Supplement: Peer review history

# Peer review: Decision Letter 0

**DOI:** 10.1371/journal.pone.0331651.r001

**Authors:** Giovanni Giordano

1 Jun 2025

Dear Dr. Leclair,

Thank you for submitting your manuscript to PLOS ONE. After careful consideration, we feel that it has merit but does not fully meet PLOS ONE’s publication criteria as it currently stands. Therefore, we invite you to submit a revised version of the manuscript that addresses the points raised during the review process.

Please submit your revised manuscript by Jul 16 2025 11:59PM. If you will need more time than this to complete your revisions, please reply to this message or contact the journal office at plosone@plos.org . When you're ready to submit your revision, log on to https://www.editorialmanager.com/pone/ and select the 'Submissions Needing Revision' folder to locate your manuscript file.

- A rebuttal letter that responds to each point raised by the academic editor and reviewer(s). You should upload this letter as a separate file labeled 'Response to Reviewers'.
- A marked-up copy of your manuscript that highlights changes made to the original version. You should upload this as a separate file labeled 'Revised Manuscript with Track Changes'.
- An unmarked version of your revised paper without tracked changes. You should upload this as a separate file labeled 'Manuscript'.

If you would like to make changes to your financial disclosure, please include your updated statement in your cover letter. Guidelines for resubmitting your figure files are available below the reviewer comments at the end of this letter.

If applicable, we recommend that you deposit your laboratory protocols in protocols.io to enhance the reproducibility of your results. Protocols.io assigns your protocol its own identifier (DOI) so that it can be cited independently in the future. For instructions see: https://journals.plos.org/plosone/s/submission-guidelines#loc-laboratory-protocols . Additionally, PLOS ONE offers an option for publishing peer-reviewed Lab Protocol articles, which describe protocols hosted on protocols.io. Read more information on sharing protocols at https://plos.org/protocols?utm_medium=editorial-email&utm_source=authorletters&utm_campaign=protocols .

We look forward to receiving your revised manuscript.

Kind regards,

Giovanni Giordano

Academic Editor

PLOS ONE

Journal Requirements:

When submitting your revision, we need you to address these additional requirements.

1. Please ensure that your manuscript meets PLOS ONE's style requirements, including those for file naming. The PLOS ONE style templates can be found at https://journals.plos.org/plosone/s/file?id=wjVg/PLOSOne_formatting_sample_main_body.pdf and https://journals.plos.org/plosone/s/file?id=ba62/PLOSOne_formatting_sample_title_authors_affiliations.pdf

2. We note that the grant information you provided in the ‘Funding Information’ and ‘Financial Disclosure’ sections do not match.

When you resubmit, please ensure that you provide the correct grant numbers for the awards you received for your study in the ‘Funding Information’ section.

3. Thank you for stating the following financial disclosure:

Funding #RQM00158 from Médicament Québec

Please state what role the funders took in the study. If the funders had no role, please state: "The funders had no role in study design, data collection and analysis, decision to publish, or preparation of the manuscript."

If this statement is not correct you must amend it as needed.

Please include this amended Role of Funder statement in your cover letter; we will change the online submission form on your behalf.

4. Thank you for stating the following in the Acknowledgments Section of your manuscript:

We are thankful to Laurent Vinet, Vanessa Kairouz and André Charette from the Department of Chemistry of University of Montreal for the supply in propofol. We are also thankful for the funding #RQM00158 from Médicament Québec.

We note that you have provided funding information that is not currently declared in your Funding Statement. However, funding information should not appear in the Acknowledgments section or other areas of your manuscript. We will only publish funding information present in the Funding Statement section of the online submission form.

Please remove any funding-related text from the manuscript and let us know how you would like to update your Funding Statement. Currently, your Funding Statement reads as follows:

Funding #RQM00158 from Médicament Québec

Please include your amended statements within your cover letter; we will change the online submission form on your behalf.

5. Please update your submission to use the PLOS LaTeX template. The template and more information on our requirements for LaTeX submissions can be found at http://journals.plos.org/plosone/s/latex.

6. Please include captions for your Supporting Information files at the end of your manuscript, and update any in-text citations to match accordingly. Please see our Supporting Information guidelines for more information: http://journals.plos.org/plosone/s/supporting-information.

[Note: HTML markup is below. Please do not edit.]

Reviewers' comments:

Reviewer's Responses to Questions

**Comments to the Author**

1. Is the manuscript technically sound, and do the data support the conclusions?

Reviewer #1: Yes

Reviewer #2: Partly

Reviewer #3: Yes

**********

2. Has the statistical analysis been performed appropriately and rigorously?

Reviewer #1: Yes

Reviewer #2: No

Reviewer #3: Yes

**********

3. Have the authors made all data underlying the findings in their manuscript fully available?

The PLOS Data policy

Reviewer #1: Yes

Reviewer #2: Yes

Reviewer #3: Yes

**********

4. Is the manuscript presented in an intelligible fashion and written in standard English?

Reviewer #1: Yes

Reviewer #2: Yes

Reviewer #3: Yes

**********

Reviewer #1: 1. The manuscript proposes that Lipocalin-2 (Lcn2) deficiency exacerbates ileitis via effects on the microbiota and mucosal immunity. While the rationale is interesting and relevant, the hypothesis needs to be better structured and clearly articulated in the introduction. A concise paragraph that lays out the working model at the end of the introduction would significantly enhance readability.

2. The 16S rRNA sequencing results are central to the study. However, key details on sequencing depth, controls, and statistical robustness are missing. For instance, rarefaction curves or measures of sequencing coverage (e.g., Good’s coverage) are not discussed. Additionally, the choice of alpha- and beta-diversity metrics needs justification. Include more robust statistical validation for LEfSe results.

3. Some bar plots lack error bars (e.g., in microbiota composition figures). Also, legends should include sample sizes (n) and significance indicators.

4. While generally readable, the manuscript contains grammatical errors and awkward phrasing (e.g., "Lcn2 knockout mice are more prone to intestinal inflammation due to defective immune"). Consider professional English editing

5. Some abbreviations (e.g., FMT, DSS, LP) are used before being defined.

However, the conclusions are not fully supported by the current data presentation and lack mechanistic depth. With significant revisions, especially in clarifying experimental design, statistical analysis, and integrating functional insight, the manuscript could become a valuable contribution to the field.

Reviewer #2: In the provided manuscript, it has been suggested that it may be possible to distribute vials containing only pure propofol to hospitals as a back-up in the event of commercial propofol emulsions running out. Reconstitution can then be carried out by a hospital pharmacist using a predefined volume of Intralipid 20% or SMOFlipid 20%, depending on what is available. Bacterial endotoxins and sterility tests should be performed before distributing such vials in hospitals which in my view is a critical challenge and risk for the overall usability of this alternative product. It would be strongly recommended to evaluate and demonstrate the shelf life of reconstituted propofol emulsions with long term chemical stability as well as for sterility against microbial growth and contamination.

The number of samples (n) tested in the study is too low to carry out any meaningful statistical analysis across multiple experiments. it would be strongly recommended to add more samples to the experimental studies and carry out statistical analysis to further support the reliability and reproducibility of the data.

Reviewer #3: Methodological Clarifications and Recommendations for Improvement

The manuscript titled "Propofol emulsification in Intralipid and SMOFlipid: a promising alternative in response to future shortages" presents an interesting and timely approach. However, to enhance scientific rigor, reproducibility, and alignment with best practices, the following issues must be addressed prior to consideration for publication:

1. Preparation Methodology:

The authors mention that emulsification was achieved through “gentle mixing,” but no further methodological details regarding time etc. are provided. It is essential to specify whether shaking was performed manually or using a mechanical device (e.g., vortex mixer or orbital shaker). If mechanical mixing was used, details such as equipment type, angle (if applicable), speed (rpm), and duration must be included.

This information is critical for ensuring reproducibility and for interpreting the consistency and quality of the emulsion.

2. Drug Extraction for HPLC Analysis:

While the extraction solvent is mentioned, the manuscript does not provide sufficient detail on the extraction procedure. The authors should clarify:

� The number of serial extraction steps performed to ensure complete recovery of Propofol

� The volume of solvent used per step

� Whether the extracts were pooled or analyzed individually

� Data supporting extraction efficiency, if any

These details are important for the reliability of quantitative results and should conform to ICH Q2(R1) requirements for method validation.

3. HPLC Method Parameters and System Suitability:

The manuscript outlines the main chromatographic conditions; however, key validation parameters required by USP and ICH guidelines are missing. The authors should include:

� System suitability data, including tailing factor, resolution (if applicable), theoretical plate count, and %RSD for retention time and peak area

� Method validation data, such as linearity, precision, accuracy, LOD/LOQ, only in table form (authors may be planning to submit a separate manuscript for HPLC -Analytical Method Development and Validation).

4. Visual Representation:

To enhance clarity and facilitate reproducibility, the manuscript would benefit from the inclusion of photographs or schematic diagrams of:

� The emulsion preparation process

� Visual appearance of stability samples at various time points

� Pictures of Output of results generated by equipment, if available.

These additions can aid in interpretation and provide qualitative support to the analytical findings.

5. Particle Size Distribution and Zeta Potential:

Given that the study involves emulsified formulations, characterization of physical stability is essential. It is recommended that the authors include:

� Particle size distribution (e.g., mean droplet diameter, polydispersity index) using dynamic light scattering or an equivalent technique

� Zeta potential measurements to assess electrostatic stability (ideally ±30 mV for stable emulsions)

� Stability data for these parameters over time to support conclusions regarding formulation robustness

This is in line with best practices and regulatory expectations (Health Canada /EMA/FDA) for injectable emulsions.

6. Check for spelling -International Conference on Harmonisation-ICH

The study addresses an important formulation challenge; however, the above methodological gaps need to be rectified to meet the standards of scientific rigor and regulatory compliance. I recommend revision of the manuscript incorporating these detailed improvements.

**********

what does this mean? ). If published, this will include your full peer review and any attached files.

If you choose “no”, your identity will remain anonymous but your review may still be made public.

**Do you want your identity to be public for this peer review?** For information about this choice, including consent withdrawal, please see our Privacy Policy

Reviewer #1: No

Reviewer #2: No

Reviewer #3: **Yes: ** Mahvash Ansari

**********

[NOTE: If reviewer comments were submitted as an attachment file, they will be attached to this email and accessible via the submission site. Please log into your account, locate the manuscript record, and check for the action link "View Attachments". If this link does not appear, there are no attachment files.]

While revising your submission, please upload your figure files to the Preflight Analysis and Conversion Engine (PACE) digital diagnostic tool, https://pacev2.apexcovantage.com/ . PACE helps ensure that figures meet PLOS requirements. To use PACE, you must first register as a user. Registration is free. Then, login and navigate to the UPLOAD tab, where you will find detailed instructions on how to use the tool. If you encounter any issues or have any questions when using PACE, please email PLOS at figures@plos.org . Please note that Supporting Information files do not need this step.

# Author comment: Author response to Decision Letter 1

**DOI:** 10.1371/journal.pone.0331651.r002

25 Jul 2025

We thank the reviewers for their time and effort. Their valuable comments have undoubtedly helped us improve the quality and clarity of this manuscript.

Reviewer #1: 1. The manuscript proposes that Lipocalin-2 (Lcn2) deficiency exacerbates ileitis via effects on microbiota and mucosal immunity. While the rationale is interesting and relevant, the hypothesis needs to be better structured and clearly articulated in the introduction. A concise paragraph that lays out the working model at the end of the introduction would significantly enhance readability.

2. The 16S rRNA sequencing results are central to the study. However, key details on sequencing depth, controls, and statistical robustness are missing. For instance, rarefaction curves or measures of sequencing coverage (e.g., Good’s coverage) are not discussed. Additionally, the choice of alpha- and beta-diversity metrics needs justification. Include more robust statistical validation for LEfSe results.

3. Some bar plots lack error bars (e.g., in microbiota composition figures). Also, legends should include sample sizes (n) and significance indicators.

4. While generally readable, the manuscript contains grammatical errors and awkward phrasing (e.g., "Lcn2 knockout mice are more prone to intestinal inflammation due to defective immune"). Consider professional English editing

5. Some abbreviations (e.g., FMT, DSS, LP) are used before being defined.

However, the conclusions are not fully supported by the current data presentation and lack mechanistic depth. With significant revisions, especially in clarifying experimental design, statistical analysis, and integrating functional insight, the manuscript could become a valuable contribution to the field.

We thank the reviewer for agreeing to revise our manuscript. However, the comments provided appear to pertain to a different subject than the one addressed in our work. As such, we believe these comments fall outside the scope of our manuscript and have not incorporated them into our revision.

Reviewer #2: In the provided manuscript, it has been suggested that it may be possible to distribute vials containing only pure propofol to hospitals as a back-up in the event of commercial propofol emulsions running out. Reconstitution can then be carried out by a hospital pharmacist using a predefined volume of Intralipid 20% or SMOFlipid 20%, depending on what is available. Bacterial endotoxins and sterility tests should be performed before distributing such vials in hospitals which in my view is a critical challenge and risk for the overall usability of this alternative product. It would be strongly recommended to evaluate and demonstrate the shelf life of reconstituted propofol emulsions with long term chemical stability as well as for sterility against microbial growth and contamination.

We thank the reviewer for this thoughtful comment regarding the sterility and stability of the proposed reconstituted propofol emulsions.

We would like to clarify that the approach we describe is intended strictly as a backup solution, to be employed only in the event of a critical shortage of commercially available propofol emulsions. Under such exceptional circumstances, the formulation would be prepared by a hospital pharmacist and administered shortly thereafter—within minutes to a few hours—thus minimizing concerns related to microbial contamination or endotoxin formation.

Importantly, the vials distributed to hospitals would contain only sterile, pure propofol, a substance with a well-established chemical stability profile, similar to reference standards obtained from certified suppliers (e.g., Toronto Research Chemicals). In cases where the vials cannot be used promptly, storage at −20 °C would further ensure both chemical stability and sterility, as propofol solidifies under these conditions, effectively limiting microbial growth and endotoxin development.

Regarding the reconstituted emulsions, we have already demonstrated chemical stability for up to 7 days. However, we emphasize that in the scenario we envision, the emulsion would be prepared and administered on the same day, further reducing any practical relevance of long-term stability or sterility concerns.

Given the emergency-use context, the immediate-use timeline, and the existing data on stability, we believe that additional long-term sterility and endotoxin studies, while certainly valuable under other circumstances, are not strictly necessary for the specific purpose and usage scenario we are addressing.

The number of samples (n) tested in the study is too low to carry out any meaningful statistical analysis across multiple experiments. it would be strongly recommended to add more samples to the experimental studies and carry out statistical analysis to further support the reliability and reproducibility of the data.

We appreciate the reviewer’s suggestion regarding the use of a larger number of samples and more extensive statistical analysis.

However, the number of replicates used in our study (n = 3 per condition, across 4 conditions) is in line with current regulatory guidelines for stability studies of compounded formulations. This approach reflects established practice and is considered sufficient by agencies such as the FDA and EMA to assess the reproducibility and reliability of results in this context.

Our results showed consistent and reproducible outcomes across replicates, which supports the robustness of the formulation under the tested conditions. While larger sample sizes and formal statistical tests can be valuable in broader pharmacological or clinical studies, they are not typically required—or expected—for stability testing in pharmaceutical compounding, where the focus is on observing trends and verifying reproducibility under defined storage conditions.

We hope this clarification addresses the reviewer’s concern.

Reviewer #3: Methodological Clarifications and Recommendations for Improvement

The manuscript titled "Propofol emulsification in Intralipid and SMOFlipid: a promising alternative in response to future shortages" presents an interesting and timely approach. However, to enhance scientific rigor, reproducibility, and alignment with best practices, the following issues must be addressed prior to consideration for publication:

1. Preparation Methodology:

The authors mention that emulsification was achieved through “gentle mixing,” but no further methodological details regarding time etc. are provided. It is essential to specify whether shaking was performed manually or using a mechanical device (e.g., vortex mixer or orbital shaker). If mechanical mixing was used, details such as equipment type, angle (if applicable), speed (rpm), and duration must be included. This information is critical for ensuring reproducibility and for interpreting the consistency and quality of the emulsion.

We thank the reviewer for highlighting the importance of methodological transparency.

In response, we have added two detailed paragraphs to the section “Propofol Compounded Formulation Preparation” (beginning at line 119 of the revised manuscript). These additions describe the manual shaking process used to achieve emulsification, including all relevant parameters such as duration, intensity, and technique.

We believe this additional information now ensures full reproducibility and addresses the reviewer’s concern regarding preparation consistency and emulsion quality.

2. Drug Extraction for HPLC Analysis:

While the extraction solvent is mentioned, the manuscript does not provide sufficient detail on the extraction procedure. The authors should clarify:

The number of serial extraction steps performed to ensure complete recovery of Propofol; The volume of solvent used per step; Whether the extracts were pooled or analyzed individually; Data supporting extraction efficiency, if any.

These details are important for the reliability of quantitative results and should conform to ICH Q2(R1) requirements for method validation.

We appreciate the reviewer’s attention to the extraction methodology, which is indeed crucial for ensuring the reliability and reproducibility of quantitative results.

In response, we have added the requested details in the two “Propofol Extraction” subsections, starting at line 248 of the revised manuscript. Specifically, we now clarify that:

• A single extraction step was sufficient to achieve near-complete recovery (~100 ± 0.3%) for Propofol in SMOFlipid preparations.

• The extraction was performed using 9.9 mL of solvent added to 100 µL of propofol emulsion, for a total volume of 10 mL.

• Extracts were analyzed individually, not pooled.

• Recovery data are provided, demonstrating that the method yields consistent and quantitative extraction of propofol, in line with expectations for analytical accuracy.

We believe these additions bring the procedure into alignment with best practices and the expectations outlined in ICH Q2(R1) for analytical method validation.

3. HPLC Method Parameters and System Suitability:

The manuscript outlines the main chromatographic conditions; however, key validation parameters required by USP and ICH guidelines are missing. The authors should include:

- System suitability data, including tailing factor, resolution (if applicable), theoretical plate count, and %RSD for retention time and peak area

- Method validation data, such as linearity, precision, accuracy, LOD/LOQ, only in table form (authors may be planning to submit a separate manuscript for HPLC -Analytical Method Development and Validation).

We thank the reviewer for raising this important point regarding method validation in accordance with USP and ICH guidelines.

A complete validation of the HPLC method—including system suitability parameters (e.g., tailing factor, theoretical plate count, %RSD for retention time and peak area) as well as method validation data (linearity, precision, accuracy, specificity)—has been provided in the Supporting Information of the manuscript.

We made the choice to include this information as supplementary material to maintain the focus of the main text on the practical and pharmaceutical aspects of the study, which we believe are of primary interest to medical professionals and compounding pharmacists. Nonetheless, we agree that these validation details are critical for ensuring analytical reliability and transparency, which is why they are fully documented and accessible in the supporting files.

4. Visual Representation:

To enhance clarity and facilitate reproducibility, the manuscript would benefit from the inclusion of photographs or schematic diagrams of:

- The emulsion preparation process

- Visual appearance of stability samples at various time points

- Pictures of Output of results generated by equipment, if available.

These additions can aid in interpretation and provide qualitative support to the analytical findings.

We thank the reviewer for this constructive suggestion.

While we initially questioned the added value of including visuals for a process as simple as combining propofol with a lipid emulsion in a vial, we agree that visual representation can help support clarity and reproducibility. As such, we have included photographs of an emulsion preparations at Day 0 and Day 7 in the Supporting Information to illustrate the visual appearance over time.

Regarding analytical output visuals, we would like to point out that we have already included:

• A representative particle size distribution graph from the LS Coulter (Figure S1)

• A chromatogram from the specificity validation of the HPLC method (Figure S3)

These visual elements, already present in the Supporting Information, contribute to the interpretation and qualitative assessment of both formulation stability and analytical performance.

We hope these additions fully address the reviewer’s recommendation.

5. Particle Size Distribution and Zeta Potential:

Given that the study involves emulsified formulations, characterization of physical stability is essential. It is recommended that the authors include:

- Particle size distribution (e.g., mean droplet diameter, polydispersity index) using dynamic light scattering or an equivalent technique

- Zeta potential measurements to assess electrostatic stability (ideally ±30 mV for stable emulsions)

- Stability data for these parameters over time to support conclusions regarding formulation robustness

This is in line with best practices and regulatory expectations (Health Canada /EMA/FDA) for injectable emulsions.

We thank the reviewer for highlighting the importance of physical characterization in evaluating emulsion stability.

Particle size distribution data have been included in the Supporting Information (Figure S1 and Table S1). These were obtained using laser diffraction with an LS Coulter instrument, a technique recognized by USP <729> as a valid and widely accepted method for evaluating mean droplet size in lipid injectable emulsions. While parameters such as polydispersity index (PDI) are informative, they are not required by regulatory agencies (e.g., Health Canada, EMA, FDA) for compounded emulsions.

Our study employed Intralipid and SMOFlipid, both of which are already approved and marketed lipid emulsions that conform to stringent regulatory standards for particle size and stability. Our results demonstrate that the addition of propofol does not significantly alter the particle size distribution of these emulsions, supporting the suitability of the compounded formulation for parenteral administration.

Regarding zeta potential, we acknowledge that this parameter can provide useful insights into electrostatic stability. However, our laser diffraction setup does not support this measurement, and such equipment is far less commonly available in hospital or compounding settings. Nonetheless, the consistency of the particle size profiles over the full duration of the stability study strongly supports the physical stability of the emulsions. These data are presented in Table 5 of the main manuscript and in the Supporting Information (Figure S1 and Table S1, Appendix 1).

In summary, while zeta potential could further support our findings, we believe that the presented data are in line with current regulatory expectations for injectable emulsions and adequately support the physical robustness of the formulations.

6. Check for spelling -International Conference on Harmonisation-ICH

Thank you for pointing this out. The spelling of International Conference on Harmonisation (ICH) has been reviewed and corrected throughout the manuscript.

The study addresses an important formulation challenge; however, the above methodological gaps need to be rectified to meet the standards of scientific rigor and regulatory compliance. I recommend revision of the manuscript incorporating these detailed improvements.

We sincerely thank the reviewer for recognizing the relevance of our work in addressing an important formulation challenge. We also appreciate the detailed and constructive feedback aimed at strengthening the methodological rigor and regulatory alignment of our study.

In response, we have carefully revised the manuscript to address each of the points raised. We believe these changes have significantly improved the clarity, reproducibility, and scientific robustness of the work, and we hope the revised version meets the standards expected for publication.

Attachment Submitted filename: Response to Reviewers.docx

# Peer review: Decision Letter 1

**DOI:** 10.1371/journal.pone.0331651.r003

**Authors:** Giovanni Giordano

20 Aug 2025

Propofol emulsification in Intralipid and SMOFlipid: a promising alternative in response to future shortages

PONE-D-25-20660R1

Dear Dr. Leclair,

We’re pleased to inform you that your manuscript has been judged scientifically suitable for publication and will be formally accepted for publication once it meets all outstanding technical requirements.

Within one week, you’ll receive an e-mail detailing the required amendments. When these have been addressed, you’ll receive a formal acceptance letter and your manuscript will be scheduled for publication.

An invoice will be generated when your article is formally accepted. Please note, if your institution has a publishing partnership with PLOS and your article meets the relevant criteria, all or part of your publication costs will be covered. Please make sure your user information is up-to-date by logging into Editorial Manager at Editorial Manager®  and clicking the ‘Update My Information' link at the top of the page. For questions related to billing, please contact billing support .

If your institution or institutions have a press office, please notify them about your upcoming paper to help maximize its impact. If they’ll be preparing press materials, please inform our press team as soon as possible -- no later than 48 hours after receiving the formal acceptance. Your manuscript will remain under strict press embargo until 2 pm Eastern Time on the date of publication. For more information, please contact onepress@plos.org.

Kind regards,

Giovanni Giordano

Academic Editor

PLOS ONE

Additional Editor Comments (optional):

Reviewers' comments:

Reviewer's Responses to Questions

**Comments to the Author**

Reviewer #1: All comments have been addressed

Reviewer #2: All comments have been addressed

Reviewer #3: All comments have been addressed

**********

2. Is the manuscript technically sound, and do the data support the conclusions?

Reviewer #1: Yes

Reviewer #2: Yes

Reviewer #3: Yes

**********

3. Has the statistical analysis been performed appropriately and rigorously?

Reviewer #1: Yes

Reviewer #2: I Don't Know

Reviewer #3: Yes

**********

4. Have the authors made all data underlying the findings in their manuscript fully available?

The PLOS Data policy

Reviewer #1: Yes

Reviewer #2: Yes

Reviewer #3: Yes

**********

5. Is the manuscript presented in an intelligible fashion and written in standard English?

Reviewer #1: Yes

Reviewer #2: Yes

Reviewer #3: Yes

**********

Reviewer #1: The manuscript is well-structured, clearly written, and presents original findings that contribute meaningfully to the field. The methodology is sound, the results are presented with clarity, and the discussion is supported by relevant literature. The authors have addressed all previous concerns satisfactorily, and the overall quality of the work meets the standards expected by the journal. Therefore, I recommend this paper for publication.

Reviewer #2: (No Response)

Reviewer #3: The authors have responded to all the comments or provided justification for the one which has not been addressed.

**********

what does this mean? ). If published, this will include your full peer review and any attached files.

If you choose “no”, your identity will remain anonymous but your review may still be made public.

**Do you want your identity to be public for this peer review?** For information about this choice, including consent withdrawal, please see our Privacy Policy

Reviewer #1: **Yes: ** Faizan Akram

Reviewer #2: No

Reviewer #3: **Yes: ** Mahvash Ansari

**********

# Editor report: Acceptance letter

**DOI:** 10.1371/journal.pone.0331651.r004

**Authors:** Giovanni Giordano

PONE-D-25-20660R1

PLOS ONE

Dear Dr. Leclair,

I'm pleased to inform you that your manuscript has been deemed suitable for publication in PLOS ONE. Congratulations! Your manuscript is now being handed over to our production team.

At this stage, our production department will prepare your paper for publication. This includes ensuring the following:

* All references, tables, and figures are properly cited

* All relevant supporting information is included in the manuscript submission,

* There are no issues that prevent the paper from being properly typeset

You will receive further instructions from the production team, including instructions on how to review your proof when it is ready. Please keep in mind that we are working through a large volume of accepted articles, so please give us a few days to review your paper and let you know the next and final steps.

Lastly, if your institution or institutions have a press office, please let them know about your upcoming paper now to help maximize its impact. If they'll be preparing press materials, please inform our press team within the next 48 hours. Your manuscript will remain under strict press embargo until 2 pm Eastern Time on the date of publication. For more information, please contact onepress@plos.org.

You will receive an invoice from PLOS for your publication fee after your manuscript has reached the completed accept phase. If you receive an email requesting payment before acceptance or for any other service, this may be a phishing scheme. Learn how to identify phishing emails and protect your accounts at https://explore.plos.org/phishing.

If we can help with anything else, please email us at customercare@plos.org.

Thank you for submitting your work to PLOS ONE and supporting open access.

Kind regards,

PLOS ONE Editorial Office Staff

on behalf of

Dr. Giovanni Giordano

Academic Editor

PLOS ONE
